# A key genomic subtype associated with lymphovascular invasion in invasive breast cancer

**DOI:** 10.1038/s41416-019-0486-6

**Published:** 2019-05-22

**Authors:** Sasagu Kurozumi, Chitra Joseph, Sultan Sonbul, Sami Alsaeed, Yousif Kariri, Abrar Aljohani, Sara Raafat, Mansour Alsaleem, Angela Ogden, Simon J Johnston, Mohammed A Aleskandarany, Takaaki Fujii, Ken Shirabe, Carlos Caldas, Ibraheem Ashankyty, Leslie Dalton, Ian O Ellis, Christine Desmedt, Andrew R Green, Nigel P Mongan, Emad A Rakha

**Affiliations:** 1 Nottingham Breast Cancer Research Centre, Division of Cancer and Stem Cells, School of Medicine, University of Nottingham, Nottingham, UK; 2 Department of General Surgical Science, Gunma University Graduate School of Medicine, Gunma, Japan; 3 Faculty of Medicine, Menoufyia University, Shebin al Kawm, Egypt; 4 Cancer Research UK Cambridge Institute and Department of Oncology, University of Cambridge, Cambridge, UK; 5 Faculty of Applied Medical Sciences, King Abdulaziz University, Jeddah, Saudi Arabia; 6 Department of Histopathology, St. David’s South Austin Medical Center, Austin, TX USA; 7 Laboratory for Translational Breast Cancer Research, Department of Oncology, KU Leuven, Leuven, Belgium; 8 Biology and Translational Research, Faculty of Medicine and Health Sciences, University of Nottingham, Nottingham, UK; 9 Department of Pharmacology, Weill Cornell Medicine, New York, NY USA

**Keywords:** Breast cancer, Breast cancer, Cancer genomics

## Abstract

**Background:**

Lymphovascular invasion (LVI) is associated with the development of metastasis in invasive breast cancer (BC). However, the complex molecular mechanisms of LVI, which overlap with other oncogenic pathways, remain unclear. This study, using available large transcriptomic datasets, aims to identify genes associated with LVI in early-stage BC patients.

**Methods:**

Gene expression data from the Molecular Taxonomy of Breast Cancer International Consortium (METABRIC) cohort (*n* = 1565) was used as a discovery dataset, and The Cancer Genome Atlas (TCGA; *n* = 854) cohort was used as a validation dataset. Key genes were identified on the basis of differential mRNA expression with respect to LVI status as characterised by histological review. The relationships among LVI-associated genomic subtype, clinicopathological features and patient outcomes were explored.

**Results:**

A 99-gene set was identified that demonstrated significantly different expression between LVI-positive and LVI-negative cases. Clustering analysis with this gene set further divided cases into two molecular subtypes (subtypes 1 and 2), which were significantly associated with pathology-determined LVI status in both cohorts. The 10-year overall survival of subtype 2 was significantly worse than that of subtype 1.

**Conclusion:**

This study demonstrates that LVI in BC is associated with a specific transcriptomic profile with potential prognostic value.

## Background

Outcomes for early-stage breast cancer (BC) patients have improved over recent decades as a result of better diagnostic accuracy, targeted drug therapies, in addition to improvements in early diagnosis.^1^ However, the ten-year mortality rates of BC patients remain ~20% which is attributable to the development of metastasis.^2^ Several histopathological features have been studied as prognostic factors in BC, including tumour size, lymph node status and histological grade,^3–5^ which are strongly associated with outcome. Lymphovascular invasion (LVI) is an early event in the development of metastasis and is a potent prognostic factor.^6^ Although the molecular profiles associated with tumour differentiation in terms of histological type and grade and development of lymph node metastasis have been well characterised,^7–9^ the molecular mechanisms of LVI and associated genes that may represent therapeutic targets or biomarkers remain to be identified. The main challenge in determining the molecular profiles associated with LVI status in BC stems from the lack of LVI status in the available large-scale molecular studies in addition to the inherent subjectivity of morphological assessment of LVI status.

The Molecular Taxonomy of Breast Cancer International Consortium (METABRIC)^10^ and The Cancer Genome Atlas (TCGA)^11^ cohorts are currently the largest genomic and transcriptomic datasets of early-stage BC patients with clinical follow-up. In this study, using these large transcriptomic datasets combined with thorough histological assessment of LVI, we applied bioinformatic analysis to evaluate the genes associated with LVI and assessed the prognostic value of genomic subtype based on LVI status.

## Methods

### The METABRIC cohort

In the METABRIC study,^10^ mRNA was extracted from primary tumours of female patients, and mRNA expression was evaluated using the Illumina TotalPrep RNA Amplification Kit and Illumina Human HT-12 v3 Expression BeadChips (Ambion, Warrington, UK). LVI status of 1565 patients within the METABRIC cohort, which were histologically assessed using haematoxylin and eosin (H&E) stained slides. For the Nottingham subset included in METABRIC (*n* = 285/1565), LVI status was additionally assessed by immunohistochemistry (IHC) utilising CD31, CD34 and D2-40,^12^ and the final LVI status was confirmed using a combination of multiple H&E tumour sections and IHC. Considering the different methods of LVI assessment, cases were divided into two groups: (1) the Nottingham cases and (2) the remaining METABRIC cases (*n* = 1280). Gene transcript expression levels between LVI-positive and LVI-negative cases were compared for each group, as described in the ‘Bioinformatics analysis’ section.

### The TCGA cohort

The data from the TCGA^11^ cohort of female BC patients (*n* = 854) was extracted from the Genomic Data Commons Data Portal and cBioPortal website.^13,14^ Briefly, the datasets of mRNA expression from RNASeqV2 were accessed along with de-identified clinical information for several clinicopathological factors and outcomes. Digital H&E-stained slides from the TCGA_BRCA cohort were accessed via the cBioPortal website, and LVI status was quantified by an expert breast pathologist (LD).

### Bioinformatics analysis

Analysis of mRNA expression data from METABRIC has been previously described.^10^ Differentially expressed genes (DEGs) between LVI-positive and LVI-negative cases were identified using the weighted average difference (WAD) method, and the DEGs were selected according to the WAD ranking.^15,16^ Lists of the top 350 genes associated with LVI for the WAD assay in both (1) the Nottingham cases in the METABRIC cohort (*n* = 285) and (2) other METABRIC cases (*n* = 1280) are shown in Supplementary Tables 1 and 2. Overlapping DEGs between the two groups were included in the gene set associated with LVI.

The Cluster 3.0 package was used for clustering and heat map construction.^17^ Clustering analysis was performed using METABRIC data as the discovery set and validated using TCGA data as the validation set. TCGA mRNA data were log2-transformed prior to clustering analysis.

For pathway analysis, the WEB-based GEne SeT AnaLysis Toolkit (WebGestalt) was used to calculate significantly enriched gene ontologies and pathways associated with these genes.^18,19^ The false discovery rate was controlled using the Benjamini–Hochberg procedure in WebGestalt, with an adjusted-*p* < 0.01 considered statistically significant.

### Statistical analysis

Statistical analyses were conducted using IBM SPSS Statistics for Windows, version 24.0 (IBM Corp., Armonk, NY, USA). The chi-squared test was used to assess differences among several clinicopathological factors, including LVI status, tumour size, lymph node status, histological grade, oestrogen receptor (ER), progesterone receptor (PR), human epidermal growth factor 2 (HER2) and molecular subtypes, as stratified by the LVI-associated genomic subtype.

Kaplan–Meier survival curves of 10-year overall survival (OS) were plotted for the METABRIC and TCGA cohorts. The 10-year OS in this study was defined as the day of death within 10 years or the day of completing follow-up from the day of surgery. In univariate and multivariate analyses, 95% confidence intervals (CIs) were assessed using the Cox proportional hazards regression model to determine the associations between clinicopathological factors (LVI status, tumour size, lymph node status, histological grade, ER, PR and HER2), including the LVI-associated genomic subtype and prognosis.

## Results

### Clinicopathological and prognostic significance of LVI status

In the METABRIC cohort, 635/1,565 (41%) were LVI-positive and 930 (59%) were LVI-negative. The LVI-positivity rate was 41.1% (117/285) in the Nottingham cases and 40.5% (518/1,280) in the remaining METABRIC cases. In the TCGA cohort, 295/854 (35%) patients were LVI-positive and 559 (65%) were LVI-negative. In both cohorts, LVI positivity was significantly associated with large tumour size (METABRIC: *p* *<* 0.0001; TCGA: *p* *=* 0.00055), positive nodal status (METABRIC and TCGA: both *p* *<* 0.0001) and high histological grade (METABRIC and TCGA: both *p* *<* 0.0001; Supplementary Table 3).

**Fig. 1:**
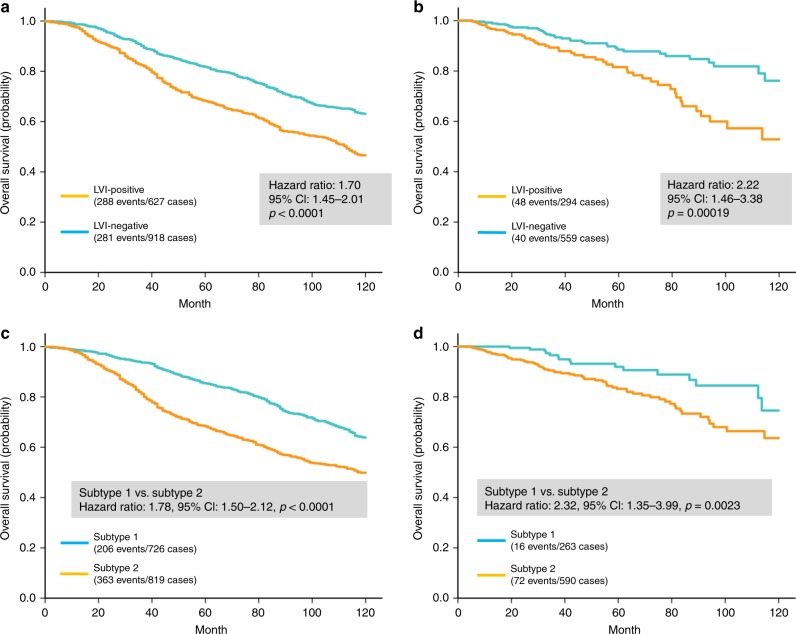
Cumulative survival of BC patients stratified by LVI status. **a** Ten-year overall survival in the METABRIC cases was significantly worse in the LVI-positive group than in the LVI-negative group. **b** In TCGA cases, significant differences were noted in patient overall survival in the LVI-positive and LVI-negative groups. Cumulative survival of breast cancer patients stratified by LVI-related genomic subtypes. **c** Ten-year overall survival in breast cancer patients with LVI-related genomic subtypes. Subtype 2 was significantly worse compared with subtype 1 in the METABRIC cohort. **d** Classification of LVI-related genomic subtype was a significant prognostic factor in the TCGA cohort

The survival of LVI-positive BC patients was significantly worse compared with LVI-negative patients in the METABRIC (hazard ratio [HR] 1.70, 95% CI 1.45–2.01, *p* *<* 0.0001; Fig. 1a) and TCGA cohorts (HR 2.2, 95% CI 1.46–3.38, *p* *=* 0.00019; Fig. 1b). Univariate and multivariate analyses of both METABRIC and TCGA datasets are summarised in Supplementary Table 4. Univariate analysis using the Cox proportional hazards regression model identified LVI-positive status, large tumour size (METABRIC: HR 1.82, 95% CI 1.49–2.21, *p* *<* 0.0001; TCGA: HR 1.81, 95% CI 1.08–3.04, *p* *=* 0.025), positive nodal status (METABRIC: HR 2.06, 95% CI 1.74–2.44, *p* *<* 0.0001; TCGA: HR 1.85, 95% CI 1.20–2.85, *p* *=* 0.0056), negative ER status (METABRIC: HR 1.66, 95% CI 1.38–1.99, *p* *<* 0.0001; TCGA: HR 1.89, 95% CI 1.19–2.98, *p* *=* 0.0065) and negative PR status (METABRIC: HR 1.67, 95% CI 1.42–1.98, *p* *<* 0.0001; TCGA: HR 1.68, 95% CI 1.08–2.61, *p* *=* 0.020) as poor prognostic factors in both cohorts. In addition, significant prognostic factors included high histological grade (HR 1.63, 95% CI 1.37–1.93, *p* *<* 0.0001) and positive HER2 status (HR 1.92, 95% CI 1.54–2.38, *p* *<* 0.0001) in the METABRIC cohort. LVI positivity was an independent poor prognostic factor in multivariate analysis (METABRIC: HR 1.29, 95% CI 1.07–1.56, *p* *=* 0.0073; TCGA: HR 2.19, 95% CI 1.32–3.62, *p* *=* 0.0023; Supplementary Table 4).

### Genes associated with LVI

The overlapping DEGs between (1) the Nottingham cases in the METABRIC cohort (*n* = 285) and (2) remaining METABRIC cases (*n* = 1280) included 42 significantly overexpressed and 57 downregulated genes (Table 1, Supplementary Tables 5 and 6).

**Table 1:** List of 99 genes significantly associated with lymphovascular invasion

Upregulated genes			Downregulated genes		
*APOC1*	*KRT7*	*UCP2*	*ACTG2*	*FCGBP*	*S100A4*
*APOE*	*KRT8*	*YWHAZ*	*ANG*	*FGD3*	*SELENOM*
*CALML5*	*LAPTM4B*		*ANXA1*	*FOS*	*SERPINA3*
*CCNB2*	*LRRC26*		*C1S*	*FST*	*SERPINE2*
*CDCA5*	*LY6E*		*CDC42EP4*	*GAS1*	*SGCE*
*COX6C*	*MMP11*		*CEBPD*	*GSTP1*	*SLC40A1*
*DNAJA4*	*MX1*		*CFB*	*HBA2*	*SLC44A1*
*EEF1A2*	*NME1*		*CFD*	*HBB*	*SRPX*
*ELF3*	*NOP56*		*CLIC6*	*HLA-DQA1*	*STC2*
*ERBB2*	*PGAP3*		*CXCL12*	*IL17RB*	*SUSD3*
*GNAS*	*PITX1*		*CXCL14*	*MAOA*	*TNS3*
*HMGA1*	*PTTG1*		*CYBRD1*	*MFAP4*	*TPM2*
*HMGB3*	*S100P*		*CYP4X1*	*MGP*	*TXNIP*
*HSPB1*	*SCD*		*DCN*	*MT1E*	*UBD*
*IDH2*	*SLC52A2*		*DKK3*	*NDP*	*VIM*
*IFI27*	*SLC9A3R1*		*DPYSL2*	*NINJ1*	*VTCN1*
*ISG15*	*SPDEF*		*DUSP1*	*PDGFRL*	*ZBTB20*
*KRT18*	*TM7SF2*		*EEF1B2*	*PLGRKT*	
*KRT18P55*	*UBE2C*		*FBLN1*	PYCARD	
*KRT19*	*UBE2S*		*FCER1A*	*RPL3*	

The 99 genes in the LVI-related set were significantly associated with gene ontologies, including ‘GO: 0005615 Extracellular space’, ‘GO: 0072562 Blood microparticle’ and ‘GO: 0031012 Extracellular matrix’ (Table 2). All significant pathways existed in the category ‘Cellular component’ of gene ontology (Supplementary Fig. 1).

**Table 2:** Gene ontology pathways significantly associated with 99 genes related to lymphovascular invasion

Ontology	Name	Genes in Ontology	Observed	Expected	Enrichment	*p*-value	Genes
GO:0005615	Extracellular space	1385	23	6.52	3.53	<0.0001	*SERPINA3, DCN, CFD, FBLN1, DKK3, ANG, GSTP1, ANXA1, HBB, HSPB1, APOC1, APOE, MFAP4, NDP, SERPINE2, S100A4, CFB, CXCL12, C1S, ACTG2, YWHAZ, STC2, CXCL14*
GO:0072562	Blood microparticle	110	7	0.52	13.51	0.00043	*SERPINA3, HBB, APOE, CFB, C1S, ACTG2, YWHAZ*
GO:0031012	Extracellular matrix	503	11	2.37	4.64	0.0079	*DCN, FBLN1, ANG, HSPB1, APOE, MFAP4, MGP, MMP11, NDP, SERPINE2, VIM*

**Fig. 2:**
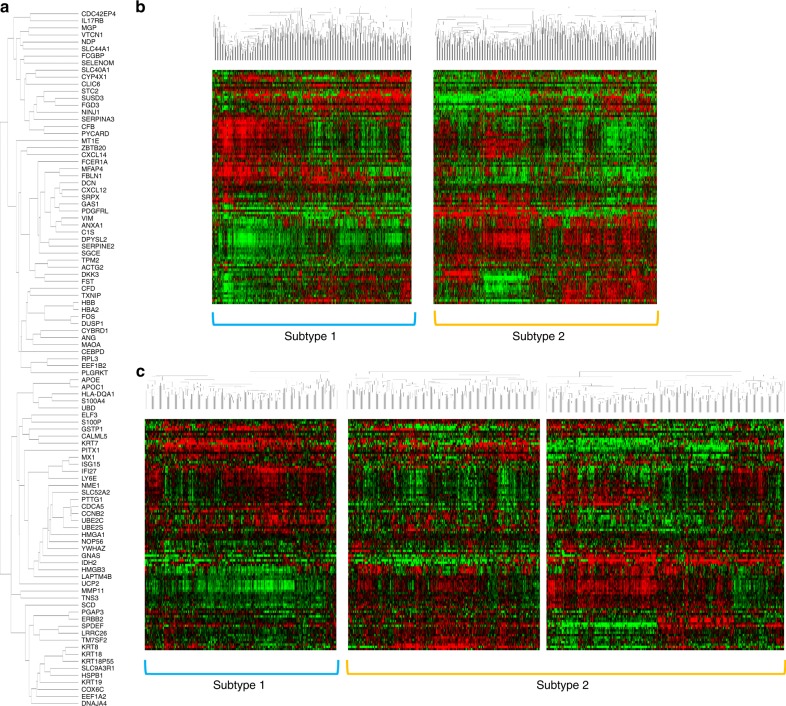
Cluster analysis of the gene set associated with LVI. **a** The dendrogram of 99 LVI-related genes using METABRIC cohort, in which the pattern of the branches indicates the relationship for each gene. Heat maps in accordance with the LVI-related gene set for the **b** METEBRIC and **c** TCGA cohorts showed that all cases were clearly divided between subtypes 1 and 2 using cluster analysis

Hierarchical clustering was used to further analyse these 99 genes based on similarity in expression (Fig. 2a). Clustering in the discovery (METABRIC) cohort classified cases into two subtypes, namely, subtypes 1 (*n* = 738 cases; 45%) and 2 (*n* = 827; 55%) (Fig. 2b). The dendrogram of METABRIC cases, in which the pattern of the branches indicates the relationship for each case, is shown in Supplementary Fig. 2.

To validate these results, hierarchical clustering was conducted on the TCGA cohort using the same 99 genes. The dendrogram classifying these 854 cases is shown in Supplementary Fig. 3, again showing the cases split into two groups: subtypes 1 and 2, with 263 (31%) and 591 (69%) cases, respectively (Fig. 2c).

In both cohorts, LVI positivity was significantly more prevalent in subtype 2 tumours than those of subtype 1 (METABRIC and TCGA: *p* *<* 0.0001; Table 3).

**Table 3:** Clinicopathological significance of genomic subtypes related to lymphovascular invasion

METABRIC cohort						TCGA cohort					
Factors		LVI-associated genomic subtypes			*p*-value	Factors		LVI-associated genomic subtypes			*p*-value
		Subtype 1	Subtype 2	Total				Subtype 1	Subtype 2	Total	
LVI	Positive	262 (35.5%)	373 (45.1%)	635	<0.0001	LVI	Positive	61 (23.2%)	234 (39.6%)	295	<0.0001
	Negative	476 (64.5%)	454 (54.9%)	930			Negative	202 (76.8%)	357 (60.4%)	559	
Tumour size	≥2 cm	454 (61.9%)	613 (75.2%)	1067	<0.0001	Tumour size	T 2–4	164 (62.4%)	451 (76.3%)	615	<0.0001
	<2 cm	279 (38.1%)	202 (24.8%)	481			T 1	99 (37.6%)	140 (23.7%)	239	
Nodal status	Positive	307 (41.7%)	428 (51.9%)	735	<0.0001	Nodal status	Positive	128 (48.9%)	295 (50.3%)	423	0.71
	Negative	429 (58.3%)	396 (48.1%)	825			Negative	134 (51.1%)	292 (49.7%)	426	
Histological grade	Grade 3	187 (26.5%)	586 (72.8%)	773	<0.0001	Histological grade	Grade 3	28 (11.3%)	324 (56.9%)	352	<0.0001
	Grade 1, 2	519 (73.5%)	219 (27.2%)	738			Grade 1, 2	219 (88.7%)	245 (43.1%)	464	
ER	Positive	707 (95.8%)	497 (60.1%)	1204	<0.0001	ER	Positive	246 (97.6%)	393 (68.7%)	185	<0.0001
	Negative	31 (4.2%)	330 (39.9%)	361			Negative	6 (2.4%)	179 (31.3%)	639	
PR	Positive	533 (72.2%)	295 (35.7%)	828	<0.0001	PR	Positive	235 (94.0%)	311 (54.8%)	546	<0.0001
	Negative	205 (27.8%)	532 (64.3%)	737			Negative	15 (6.0%)	257 (45.2%)	272	
HER2	Positive	20 (2.7%)	168 (20.3%)	188	<0.0001	HER2	Positive	20 (9.6%)	113 (23.0%)	133	<0.0001
	Negative	718 (97.3%)	659 (79.7%)	1377			Negative	189 (90.4%)	378 (77.0%)	567	
Molecular subtypes	Luminal A	467 (63.5%)	126 (15.3%)	593	<0.0001						
	Luminal B	121 (16.5%)	272 (32.9%)	393							
	HER2-enriched	10 (1.4%)	171 (20.7%)	181							
	Basal-like	24 (3.3%)	222 (26.9%)	246							
	Normal-like	113 (15.4%)	35 (4.2%)	148							

*ER* oestrogen receptor, *PR* progesterone receptor, *LVI* Lymphovascular invasion

### Clinicopathological and prognostic significance of the LVI-related gene sets

In the METABRIC and TCGA cohorts, subtype 2 was significantly associated with large tumour size (both *p* *<* 0.0001), high histological grade (both *p* *<* 0.0001), ER negativity (both *p* *<* 0.0001), PR negativity (both *p* *<* 0.0001) and HER2 positivity (both *p* *<* 0.0001; Table 3). Interestingly, 69% of luminal B, 95% HER2-enriched and 90% basal-like BC were classified as subtype 2 in the METABRIC cohort.

**Fig. 3:**
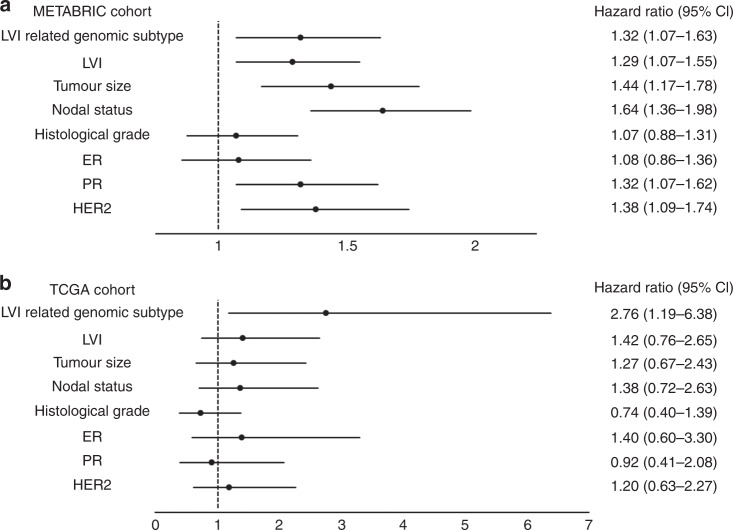
Survival analysis based on clinicopathological characteristics including LVI-related genomic subtype. Forest plots showing the hazard ratios and 95% CI of the multivariate survival analyses in **a** the METABRIC cohort and **b** the TCGA cohort. The LVI-related genomic subtype was an independent prognostic factor in both cohorts

Patients with LVI-related subtype 2 had a significantly worse prognosis compared with those presenting with subtype 1 tumours in both cohorts (METABRIC: HR 1.78, 95% CI 1.50–2.12, *p* *<* 0.0001; TCGA: HR 2.32, 95% CI 1.35–3.99, *p* *=* 0.0023; Fig. 1c, d). In multivariate survival analysis, the LVI-related genomic subtype was an independent poor prognostic factor in both cohorts (METABRIC: HR 1.32, 95% CI 1.07–1.63, *p* *=* 0.0098; TCGA: HR 2.76, 95% CI 1.19–6.38, *p* *=* 0.018; Fig. 3 and Supplementary Table 7).

## Discussion

In this study, we identified a 99-gene set significantly associated with LVI status in the METABRIC dataset. We validated this finding using the TCGA dataset. LVI is a biomarker for aggressive BC and is considered predictive for metastasis.^20^ In other cancer types, gene sets associated with vascular invasion have been previously described, for example in hepatocellular carcinoma^21^ and endometrial cancer.^22^ Mannelqvist et al.^23^ suggested that an 18-gene set associated with vascular invasion in endometrial cancer^22^ was consistently associated with hormone receptor negativity, HER2 positivity, basal-like phenotype, reduced patient survival in BC patients. In line with these findings, the present study found that 69% of luminal B, 95% HER2-enriched and 90% basal-like BCs were subtype 2 in the METABRIC cohort. Subtype 2 was significantly associated with LVI positivity. However, of the 18 genes identified in Mannelqvist et al., only different isoforms of matrix metallopeptidase (MMP) and serpin family E member (SERPINE) were present in our 99-gene set.

The underlying molecular mechanisms driving LVI in BC, which are potential therapeutic targets, have yet to be identified. The 99 genes in the LVI-related gene signature from this study are significantly associated with extracellular pathways. In previous work, Klahan et al.^24^ suggested their gene set associated with LVI was related to extracellular matrix components using microarray data from 108 BC patients. Epithelial–mesenchymal transition (EMT)-implicated genes in prostate cancer have also been associated with pathways relating to the extracellular space.^25^ The extracellular matrix comprises a network of structural proteins, and reorganisation of this matrix is required for cancer to progress.^26^ The EMT is thought to play an important role in the process of metastasis to distant sites, and certain EMT markers are related to LVI status in BC.^12^ In the 99 gene LVI signature set, there are several genes associated with extracellular pathways that are implicated in BC prognosis. For example, heat shock protein 27 (HSPB1), is associated with BC aggressiveness and metastasis.^27^ HSPB1 expression is upregulated in the early phase of cell differentiation, which implies that HSPB1 may play an important role in controlling the growth and migration of cancer stem-like cells.^28^ Another example is apolipoprotein C1 (APOC1), which is considered as a prognostic biomarker for triple-negative BC.^29^ APOC1 is thought to regulate the inflammatory response in cancer tissues,^30^ which may be closely related to the elimination of proliferating cancer cells.^31^ Upregulation of MMPs is also related to cancer cell proliferation, invasion and epithelial-to-mesenchymal transformation and is indicative of a poor prognosis for BC patients.^32^ As an example, MMP-11, which belongs to the MMP family, promotes BC development by inhibiting apoptosis as well as enhancing the migration and invasion of BC cells.^33^ Additional functional studies of these genes are necessary to explore the association of aberrant gene function and proteins related to LVI in BC.

Comparison of the METABRIC and TCGA cohorts was a limiting factor in this study, in terms of the different methods used to quantify and statistically analyse gene expression and in the approaches to LVI evaluation. We previously developed a method for the accurate detection of LVI using immunostaining for CD34 or D2-40.^12^ In the Nottingham cases, we evaluated LVI status using strict criteria based on both morphology and immunohistochemistry. However, for the TCGA BRCA cohort, we evaluated LVI status using H&E-stained slides alone from the cBioPortal database. Although LVI evaluation using only one H&E slide is feasible, it may be difficult to clearly identify LVI negativity.^34^ In present study, the LVI-positivity rates were closely similar between the Nottingham cases, the remaining METABRIC cases and TCGA_BRCA cases using the different LVI-evaluations. Although our results might suggest the adequacy of LVI evaluation with only one H&E-stained slide, further analysis with the larger cohorts to assess the LVI status using both H&E and IHC slides is necessary to report accurately on LVI status.

Microarrays were used to evaluate mRNA expression in the METABRIC analysis. In contrast, RNA-seq using NGS was used in the TCGA analysis. Microarray platforms have been used and validated for nearly two decades, and this approach has been widely used for evaluating multi-gene expression. Conversely, the unbiased genome-wide RNA-seq method allows for the analysis of all annotated transcripts in addition to the identification of novel transcripts, splice junctions and noncoding RNAs. These technological and methodological differences may underpin the known challenges of relating microarray and RNA sequencing data between studies.^35,36^ For example, the different approaches can have different lower limits of detection or may encompass different genomic regions. Thus, we cannot assume that the methods are interchangeable, and doing so would require rigorous cross-assay comparisons.^37^ Although there is statistical agreement across the different cohorts in the present study, further analysis using identical technologies (microarray and/or NGS assays) may provide clearer validation of the LVI gene signature.

In conclusion, we have confirmed the suitability and prognostic significance of our LVI-evaluation approach using the METABRIC and TCGA cohorts. We have determined genomic subtype associated with LVI status and patient outcome in BC, therefore, providing an experimental tool which may serve to unravel the complex gene networks associated with LVI with potential clinical relevance. Consistency between clinical cohorts stratified by LVI-gene signature may be further improved by using the same definitions and evaluation methods for LVI status.

## Supplementary information

List of top 350 genes significantly associated with lymphovascular invasion in the Nottingham cohort

List of top 350 genes significantly associated with lymphovascular invasion in the remaining METABRIC cases

Correlation between lymphovascular invasion and clinicopathological characteristics

Survival analysis based on clinicopathological characteristics including lymphovascular invasion

Full gene name list of the 99 genes significantly associated with lymphovascular invasion

Mean value, standard error of the mean (SEM), subtraction and weighted average difference (WAD) ranking in the 99 genes significantly associated with lymphovascular invasion

Survival analysis based on clinicopathological characteristics including LVI-related genomic subtype

Significant pathways associated with LVI-related gene set

The dendrogram of METABRIC cases for hierarchical clustering analysis

The dendrogram of TCGA cases for hierarchical clustering analysis

## Data Availability

The datasets generated and/or analysed during the current study are available from the corresponding author on reasonable request.

## References

[1] Marshall DC, Webb TE, Hall RA, Salciccioli JD, Ali R, Maruthappu M (2016). Trends in UK regional cancer mortality 1991–2007. Br. J. Cancer.

[2] Liedtke C, Mazouni C, Hess KR, André F, Tordai A, Mejia JA (2008). Response to neoadjuvant therapy and long-term survival in patients with triple-negative breast cancer. J. Clin. Oncol..

[3] Wo JY, Chen K, Neville BA, Lin NU, Punglia RS (2011). Effect of very small tumor size on cancer-specific mortality in node-positive breast cancer. J. Clin. Oncol..

[4] Hernandez-Aya LF, Chavez-Macgregor M, Lei X, Meric-Bernstam F, Buchholz TA, Hsu L (2011). Nodal status and clinical outcomes in a large cohort of patients with triple-negative breast cancer. J. Clin. Oncol..

[5] Rakha EA, Reis-Filho JS, Baehner F, Dabbs DJ, Decker T, Eusebi V (2010). Breast cancer prognostic classification in the molecular era: the role of histological grade. Breast Cancer Res..

[6] Rakha EA, Martin S, Lee AH, Morgan D, Pharoah PD, Hodi Z (2012). The prognostic significance of lymphovascular invasion in invasive breast carcinoma. Cancer.

[7] Yates LR, Desmedt C (2017). Translational genomics: practical applications of the genomic revolution in breast cancer. Clin. Cancer Res..

[8] Sotiriou C, Wirapati P, Loi S, Harris A, Fox S, Smeds J (2006). Gene expression profiling in breast cancer: understanding the molecular basis of histologic grade to improve prognosis. J. Natl Cancer Inst..

[9] Mobadersany P, Yousefi S, Amgad M, Gutman DA, Barnholtz-Sloan JS, Velázquez Vega JE (2018). Predicting cancer outcomes from histology and genomics using convolutional networks. Proc. Natl Acad. Sci. USA.

[10] Curtis C, Shah SP, Chin SF, Turashvili G, Rueda OM, Dunning MJ (2012). The genomic and transcriptomic architecture of 2,000 breast tumours reveals novel subgroups. Nature.

[11] Ciriello G, Gatza ML, Beck AH, Wilkerson MD, Rhie SK, Pastore A (2015). Comprehensive molecular portraits of invasive lobular breast cancer. Cell.

[12] Mohammed RA, Martin SG, Mahmmod AM, Macmillan RD, Green AR, Paish EC (2011). Objective assessment of lymphatic and blood vascular invasion in lymph node-negative breast carcinoma: findings from a large case series with long-term follow-up. J. Pathol..

[13] Cerami E, Gao J, Dogrusoz U, Gross BE, Sumer SO, Aksoy BA (2012). The cBio cancer genomics portal: an open platform for exploring multidimensional cancer genomics data. Cancer Discov..

[14] Gao J, Aksoy BA, Dogrusoz U, Dresdner G, Gross B, Sumer SO (2013). Integrative analysis of complex cancer genomics and clinical profiles using the cBioPortal. Sci. Signal.

[15] Kadota K, Nakai Y, Shimizu K (2008). A weighted average difference method for detecting differentially expressed genes from microarray data. Algorithms Mol. Biol..

[16] Alexander-Dann B, Pruteanu LL, Oerton E, Sharma N, Berindan-Neagoe I, Módos D (2018). Developments in toxicogenomics: understanding and predicting compound-induced toxicity from gene expression data. Mol. Omics.

[17] De Hoon MJL, Imoto S, Nolan J, Miyano S (2004). Open source clustering software. Bioinformatics.

[18] Zhang B, Kirov S, Snoddy J (2005). WebGestalt: an integrated system for exploring gene sets in various biological contexts. Nucleic Acids Res..

[19] Wang J, Vasaikar S, Shi Z, Greer M, Zhang B (2017). WebGestalt 2017: a more comprehensive, powerful, flexible and interactive gene set enrichment analysis toolkit. Nucleic Acids Res..

[20] Aleskandarany MA, Sonbul SN, Mukherjee A, Rakha EA (2015). Molecular mechanisms underlying lymphovascular invasion in invasive breast cancer. Pathobiology.

[21] Mínguez B, Hoshida Y, Villanueva A, Toffanin S, Cabellos L, Thung S (2011). Gene-expression signature of vascular invasion in hepatocellular carcinoma. J. Hepatol..

[22] Mannelqvist M, Stefansson IM, Bredholt G, Hellem Bø T, Oyan AM, Jonassen I (2011). Gene expression patterns related to vascular invasion and aggressive features in endometrial cancer. Am. J. Pathol..

[23] Mannelqvist M, Wik E, Stefansson IM, Akslen LA (2014). An 18-gene signature for vascular invasion is associated with aggressive features and reduced survival in breast cancer. PLoS ONE.

[24] Klahan S, Wong HS, Tu SH, Chou WH, Zhang YF, Ho TF (2017). Identification of genes and pathways related to lymphovascular invasion in breast cancer patients: a bioinformatics analysis of gene expression profiles. Tumour Biol..

[25] Zhao M, Liu Y, Qu H (2016). Expression of epithelial-mesenchymal transition-related genes increases with copy number in multiple cancer types. Oncotarget.

[26] Jena MK, Janjanam J (2018). Role of extracellular matrix in breast cancer development: a brief update. F1000Res.

[27] Musiani D, Konda JD, Pavan S, Torchiaro E, Sassi F, Noghero A (2014). Heat-shock protein 27 (HSP27, HSPB1) is up-regulated by MET kinase inhibitors and confers resistance to MET-targeted therapy. FASEB J..

[28] Wei L, Liu TT, Wang HH, Hong HM, Yu AL, Feng HP (2011). Hsp27 participates in the maintenance of breast cancer stem cells through regulation of epithelial-mesenchymal transition and nuclear factor-κB. Breast Cancer Res..

[29] Song D, Yue L, Zhang J, Ma S, Zhao W, Guo F (2016). Diagnostic and prognostic significance of serum apolipoprotein C-I in triple-negative breast cancer based on mass spectrometry. Cancer Biol. Ther..

[30] Ko HL, Wang YS, Fong WL, Chi MS, Chi KH, Kao SJ (2014). Apolipoprotein C1 (APOC1) as a novel diagnostic and prognostic biomarker for lung cancer: a marker phase I trial. Thorac. Cancer.

[31] Kurozumi S, Fujii T, Matsumoto H, Inoue K, Kurosumi M, Horiguchi J (2017). Significance of evaluating tumor-infiltrating lymphocytes (TILs) and programmed cell death-ligand 1 (PD-L1) expression in breast cancer. Med. Mol. Morphol..

[32] Merdad A, Karim S, Schulten HJ, Dallol A, Buhmeida A, Al-Thubaity F (2014). Expression of matrix metalloproteinases (MMPs) in primary human breast cancer: MMP-9 as a potential biomarker for cancer invasion and metastasis. Anticancer Res..

[33] Zhang X, Huang S, Guo J, Zhou L, You L, Zhang T (2016). Insights into the distinct roles of MMP-11 in tumor biology and future therapeutics (Review). Int J. Oncol..

[34] Rakha EA, Abbas A, Pinto Ahumada P, ElSayed ME, Colman D, Pinder SE (2018). Diagnostic concordance of reporting lymphovascular invasion in breast cancer. J. Clin. Pathol..

[35] Zhao S, Fung-Leung WP, Bittner A, Ngo K, Liu X (2014). Comparison of RNA-Seq and microarray in transcriptome profiling of activated T cells. PLoS ONE.

[36] Wolff A, Bayerlová M, Gaedcke J, Kube D, Beißbarth T (2018). A comparative study of RNA-Seq and microarray data analysis on the two examples of rectal-cancer patients and Burkitt Lymphoma cells. PLoS ONE.

[37] Merker JD, Oxnard GR, Compton C, Diehn M, Hurley P, Lazar AJ (2018). Circulating tumor DNA analysis in patients with cancer: American Society of Clinical Oncology and College of American Pathologists Joint Review. J. Clin. Oncol..

