# Supplementary material for: A key genomic subtype associated with lymphovascular invasion in invasive breast cancer
Source: Br J Cancer. 2019 May 22;120(12):1129–36. doi: 10.1038/s41416-019-0486-6 (PMC6738092; doi:10.1038/s41416-019-0486-6)
Supplement: Supplementary file 1 — List of top 350 genes significantly associated with lymphovascular invasion in the Nottingham cohort [file 41416_2019_486_MOESM1_ESM.docx]

**Supplementary Table 1. List of top 350 genes significantly associated with lymphovascular invasion in** **the Nottingham cohort**

| **Gene symbols** | **WAD value** | **WAD ranking** |
| --- | --- | --- |
| ***C10orf116*** | **−0.339** | **1** |
| ***MGP*** | **−0.291** | **2** |
| ***EEF1A2*** | **0.256** | **3** |
| ***CFB*** | **−0.236** | **4** |
| ***S100P*** | **0.217** | **5** |
| ***STC2*** | **−0.217** | **6** |
| ***SUSD3*** | **−0.215** | **7** |
| ***CDH1*** | **0.212** | **8** |
| ***MX1*** | **0.209** | **9** |
| ***CFD*** | **−0.201** | **10** |
| ***PITX1*** | **0.199** | **11** |
| ***COMP*** | **−0.194** | **12** |
| ***FABP4*** | **−0.190** | **13** |
| ***C1orf64*** | **−0.187** | **14** |
| ***MT1E*** | **−0.174** | **15** |
| ***SERPINE2*** | **−0.173** | **16** |
| ***FBLN1*** | **−0.170** | **17** |
| ***PLIN4*** | **−0.169** | **18** |
| ***FCGBP*** | **−0.167** | **19** |
| ***CIDEC*** | **−0.165** | **20** |
| ***FGD3*** | **−0.164** | **21** |
| ***FASN*** | **0.164** | **22** |
| ***ESR1*** | **−0.162** | **23** |
| ***IFI27*** | **0.161** | **24** |
| ***APOC1*** | **0.157** | **25** |
| ***SCUBE2*** | **−0.153** | **26** |
| ***SLC7A5*** | **0.151** | **27** |
| ***TFF1*** | **−0.150** | **28** |
| ***LGALS1*** | **−0.150** | **29** |
| ***ALDOA*** | **0.149** | **30** |
| ***APOE*** | **0.147** | **31** |
| ***HSPB1*** | **0.146** | **32** |
| ***GSTP1*** | **−0.145** | **33** |
| ***ADH1A*** | **−0.145** | **34** |
| ***LGALS3BP*** | **0.144** | **35** |
| ***KRT18*** | **0.143** | **36** |
| ***TOMM7*** | **−0.143** | **37** |
| ***IFI6*** | **0.142** | **38** |
| ***GPX3*** | **−0.142** | **39** |
| ***UBD*** | **−0.142** | **40** |
| ***FST*** | **−0.142** | **41** |
| ***SFRP4*** | **−0.141** | **42** |
| ***SHISA2*** | **−0.141** | **43** |
| ***RPS13*** | **−0.141** | **44** |
| ***CXCL12*** | **−0.140** | **45** |
| ***SLC44A1*** | **−0.139** | **46** |
| ***AGR3*** | **−0.138** | **47** |
| ***abParts*** | **0.137** | **48** |
| ***CXCL14*** | **−0.136** | **49** |
| ***CLIC6*** | **−0.135** | **50** |
| ***PIP*** | **0.135** | **51** |
| ***TGFBR3*** | **−0.135** | **52** |
| ***C1S*** | **−0.134** | **53** |
| ***DKK3*** | **−0.134** | **54** |
| ***SELM*** | **−0.133** | **55** |
| ***DPYSL3*** | **−0.132** | **56** |
| ***VIM*** | **−0.131** | **57** |
| ***DBNDD1*** | **0.131** | **58** |
| ***SLC40A1*** | **−0.131** | **59** |
| ***MT1A*** | **−0.130** | **60** |
| ***CALML5*** | **0.129** | **61** |
| ***ACTG1*** | **0.128** | **62** |
| ***SERPINA3*** | **−0.127** | **63** |
| ***CYP4X1*** | **−0.127** | **64** |
| ***ERBB2*** | **0.125** | **65** |
| ***MDK*** | **0.124** | **66** |
| ***PDGFRL*** | **−0.123** | **67** |
| ***RPL26*** | **−0.123** | **68** |
| ***TACSTD2*** | **0.122** | **69** |
| ***UBE2S*** | **0.122** | **70** |
| ***KIAA0531*** | **−0.119** | **71** |
| ***LY6E*** | **0.118** | **72** |
| ***HBB*** | **−0.118** | **73** |
| ***RPS9*** | **−0.118** | **74** |
| ***RPS3*** | **−0.118** | **75** |
| ***S100A6*** | **−0.118** | **76** |
| ***RPS6*** | **−0.117** | **77** |
| ***EIF3E*** | **−0.116** | **78** |
| ***YWHAZ*** | **0.116** | **79** |
| ***CA12*** | **−0.116** | **80** |
| ***HLA-DPA1*** | **−0.116** | **81** |
| ***PHGDH*** | **0.115** | **82** |
| ***HSP90AA1*** | **0.115** | **83** |
| ***MAOA*** | **−0.115** | **84** |
| ***LAPTM4B*** | **0.115** | **85** |
| ***ACTB*** | **0.115** | **86** |
| ***SEZ6L2*** | **0.115** | **87** |
| ***KRT19*** | **0.115** | **88** |
| ***TFAP2A*** | **0.114** | **89** |
| ***KRT8*** | **0.114** | **90** |
| ***PRRX1*** | **−0.114** | **91** |
| ***PPP1R1B*** | **−0.113** | **92** |
| ***RPS20*** | **−0.113** | **93** |
| ***BEX1*** | **−0.113** | **94** |
| ***ANXA1*** | **−0.112** | **95** |
| ***RUSC1*** | **0.112** | **96** |
| ***IFITM1*** | **−0.111** | **97** |
| ***CAP2*** | **−0.111** | **98** |
| ***PLAC9*** | **−0.111** | **99** |
| ***ACTG2*** | **−0.111** | **100** |
| ***GNAS*** | **0.111** | **101** |
| ***RARRES2*** | **−0.111** | **102** |
| ***HLA-DRA*** | **−0.110** | **103** |
| ***RPL10A*** | **−0.110** | **104** |
| ***RPS18*** | **−0.110** | **105** |
| ***UBE2C*** | **0.110** | **106** |
| ***GLTSCR2*** | **−0.109** | **107** |
| ***SLC9A3R1*** | **0.109** | **108** |
| ***GFRA1*** | **−0.109** | **109** |
| ***CTSD*** | **0.109** | **110** |
| ***AKR1C2*** | **−0.108** | **111** |
| ***RPL3*** | **−0.108** | **112** |
| ***HSP90AB1*** | **0.107** | **113** |
| ***HSPA1A*** | **0.107** | **114** |
| ***ARHGEF6*** | **−0.107** | **115** |
| ***AKR1C3*** | **−0.107** | **116** |
| ***RPL13AP6*** | **−0.106** | **117** |
| ***LPAR1*** | **−0.106** | **118** |
| ***SOCS2*** | **−0.106** | **119** |
| ***FLJ40504*** | **0.105** | **120** |
| ***SAA1*** | **−0.105** | **121** |
| ***PYCARD*** | **−0.105** | **122** |
| ***HLA-DMA*** | **−0.105** | **123** |
| ***COL8A1*** | **−0.105** | **124** |
| ***LRRC26*** | **0.105** | **125** |
| ***MYB*** | **−0.105** | **126** |
| ***IRX3*** | **0.105** | **127** |
| ***RPS17*** | **−0.104** | **128** |
| ***TNS3*** | **−0.104** | **129** |
| ***MFAP5*** | **−0.104** | **130** |
| ***RPL24*** | **−0.103** | **131** |
| ***TM7SF2*** | **0.103** | **132** |
| ***TMSB10*** | **0.102** | **133** |
| ***VTCN1*** | **−0.102** | **134** |
| ***HIST1H2BK*** | **0.102** | **135** |
| ***AX746718*** | **−0.102** | **136** |
| ***MGST1*** | **0.102** | **137** |
| ***ADAM15*** | **0.101** | **138** |
| ***RPS14*** | **−0.100** | **139** |
| ***ISG15*** | **0.100** | **140** |
| ***MFAP4*** | **−0.099** | **141** |
| ***APOD*** | **0.099** | **142** |
| ***AZGP1*** | **0.099** | **143** |
| ***FCER1A*** | **−0.099** | **144** |
| ***MT2A*** | **−0.099** | **145** |
| ***CPB1*** | **0.099** | **146** |
| ***ATP6V1B1*** | **0.098** | **147** |
| ***S100A8*** | **0.098** | **148** |
| ***RPL21*** | **−0.098** | **149** |
| ***RPS26P11*** | **−0.098** | **150** |
| ***DPT*** | **−0.097** | **151** |
| ***NDP*** | **−0.097** | **152** |
| ***IL6ST*** | **−0.097** | **153** |
| ***SMARCA1*** | **−0.097** | **154** |
| ***PDK3*** | **−0.096** | **155** |
| ***TPST2*** | **−0.096** | **156** |
| ***GAS6*** | **−0.096** | **157** |
| ***SLC38A1*** | **−0.096** | **158** |
| ***RPL35A*** | **−0.095** | **159** |
| ***C9orf46*** | **−0.095** | **160** |
| ***PPP1R3C*** | **−0.095** | **161** |
| ***CYBRD1*** | **−0.095** | **162** |
| ***CNN3*** | **−0.095** | **163** |
| ***ITPRIPL2*** | **−0.095** | **164** |
| ***TUBA1C*** | **0.094** | **165** |
| ***HMGB3*** | **0.094** | **166** |
| ***ATP6AP1*** | **0.094** | **167** |
| ***HIST1H4C*** | **−0.093** | **168** |
| ***CSTB*** | **0.093** | **169** |
| ***RAI14*** | **−0.093** | **170** |
| ***TCEAL4*** | **−0.093** | **171** |
| ***CLDN7*** | **0.093** | **172** |
| ***PLS3*** | **−0.092** | **173** |
| ***CR610863*** | **0.092** | **174** |
| ***BTG2*** | **−0.092** | **175** |
| ***SGCE*** | **−0.092** | **176** |
| ***WBP5*** | **−0.092** | **177** |
| ***ALDH2*** | **−0.091** | **178** |
| ***IDH2*** | **0.091** | **179** |
| ***ACOX2*** | **−0.091** | **180** |
| ***SERPINF1*** | **−0.091** | **181** |
| ***CIDEA*** | **−0.091** | **182** |
| ***RPL27A*** | **−0.091** | **183** |
| ***MMP11*** | **0.090** | **184** |
| ***EFEMP1*** | **−0.090** | **185** |
| ***ANG*** | **−0.090** | **186** |
| ***CCL15*** | **−0.090** | **187** |
| ***HLA-DQA1*** | **−0.090** | **188** |
| ***UCP2*** | **0.090** | **189** |
| ***RPL36*** | **−0.089** | **190** |
| ***ECM2*** | **−0.089** | **191** |
| ***S100A9*** | **0.089** | **192** |
| ***BTG1*** | **−0.088** | **193** |
| ***C13orf15*** | **−0.088** | **194** |
| ***CITED2*** | **−0.088** | **195** |
| ***HOXB2*** | **0.088** | **196** |
| ***CDC42EP4*** | **−0.088** | **197** |
| ***CAV1*** | **−0.088** | **198** |
| ***PGAP3*** | **0.088** | **199** |
| ***SCD*** | **0.087** | **200** |
| ***FAU*** | **−0.087** | **201** |
| ***LRRC17*** | **−0.087** | **202** |
| ***PROM2*** | **0.087** | **203** |
| ***CCL5*** | **−0.087** | **204** |
| ***DNAJA4*** | **0.087** | **205** |
| ***IFITM2*** | **−0.087** | **206** |
| ***ARHGEF3*** | **−0.087** | **207** |
| ***HCST*** | **−0.087** | **208** |
| ***S100A4*** | **−0.087** | **209** |
| ***HIST1H4H*** | **0.086** | **210** |
| ***ALDH3A2*** | **−0.086** | **211** |
| ***RFTN1*** | **−0.086** | **212** |
| ***YWHAQ*** | **0.086** | **213** |
| ***DPYSL2*** | **−0.086** | **214** |
| ***RPL22*** | **−0.086** | **215** |
| ***PFKP*** | **0.085** | **216** |
| ***NME1*** | **0.085** | **217** |
| ***COMMD6*** | **−0.085** | **218** |
| ***EEF1B2*** | **−0.085** | **219** |
| ***NFKBIZ*** | **0.085** | **220** |
| ***VCAM1*** | **−0.084** | **221** |
| ***CALM1*** | **0.084** | **222** |
| ***KRT7*** | **0.084** | **223** |
| ***SLC25A5*** | **0.083** | **224** |
| ***MGC87042*** | **−0.083** | **225** |
| ***BCAP31*** | **0.083** | **226** |
| ***GAS1*** | **−0.083** | **227** |
| ***MMP9*** | **0.083** | **228** |
| ***FTL*** | **0.083** | **229** |
| ***MDH2*** | **0.082** | **230** |
| ***C8orf40*** | **−0.082** | **231** |
| ***CDCA5*** | **0.082** | **232** |
| ***GLYATL2*** | **0.082** | **233** |
| ***TPSAB1*** | **−0.082** | **234** |
| ***RNASE1*** | **0.082** | **235** |
| ***HLA-DRB6*** | **−0.081** | **236** |
| ***HLA-DQB1*** | **−0.081** | **237** |
| ***CRYAB*** | **−0.081** | **238** |
| ***CPA3*** | **−0.081** | **239** |
| ***C10orf10*** | **−0.081** | **240** |
| ***TUBB*** | **0.081** | **241** |
| ***NOP56*** | **0.081** | **242** |
| ***FERMT2*** | **−0.081** | **243** |
| ***PRKCDBP*** | **−0.080** | **244** |
| ***CD24*** | **0.080** | **245** |
| ***GRN*** | **0.080** | **246** |
| ***MXRA5*** | **−0.080** | **247** |
| ***LASP1*** | **0.080** | **248** |
| ***WISP2*** | **−0.080** | **249** |
| ***POLD2*** | **0.080** | **250** |
| ***POTEKP*** | **0.080** | **251** |
| ***ARL6IP5*** | **−0.080** | **252** |
| ***GBP2*** | **−0.080** | **253** |
| ***TSPYL5*** | **0.080** | **254** |
| ***FLNB*** | **−0.079** | **255** |
| ***H2AFY2*** | **0.079** | **256** |
| ***PTTG1*** | **0.079** | **257** |
| ***COX5A*** | **0.079** | **258** |
| ***TXNIP*** | **−0.078** | **259** |
| ***EIF3L*** | **−0.078** | **260** |
| ***ATHL1*** | **−0.078** | **261** |
| ***CHCHD2*** | **0.078** | **262** |
| ***COX6C*** | **0.078** | **263** |
| ***BCL2*** | **−0.078** | **264** |
| ***XBP1*** | **−0.078** | **265** |
| ***EPN1*** | **0.078** | **266** |
| ***FOXA1*** | **0.078** | **267** |
| ***CCND2*** | **−0.078** | **268** |
| ***LSM1*** | **−0.078** | **269** |
| ***SNAR-A3*** | **0.078** | **270** |
| ***ZNF217*** | **0.077** | **271** |
| ***RPSA*** | **−0.077** | **272** |
| ***CD36*** | **−0.077** | **273** |
| ***ELF3*** | **0.077** | **274** |
| ***TPM2*** | **−0.077** | **275** |
| ***SAPS2*** | **−0.077** | **276** |
| ***NFIB*** | **−0.077** | **277** |
| ***MBOAT7*** | **0.077** | **278** |
| ***ATP5B*** | **0.076** | **279** |
| ***C7orf41*** | **−0.076** | **280** |
| ***ABCB9*** | **0.076** | **281** |
| ***CDR2L*** | **0.076** | **282** |
| ***RPS28*** | **−0.076** | **283** |
| ***LMTK3*** | **0.076** | **284** |
| ***P4HB*** | **0.075** | **285** |
| ***ATP5C1*** | **0.075** | **286** |
| ***F13A1*** | **−0.075** | **287** |
| ***ULK1*** | **0.075** | **288** |
| ***KLHDC9*** | **−0.075** | **289** |
| ***ZG16B*** | **0.074** | **290** |
| ***TMED9*** | **0.074** | **291** |
| ***ZMIZ1*** | **0.074** | **292** |
| ***ATP2A2*** | **0.074** | **293** |
| ***RPL27*** | **−0.074** | **294** |
| ***GPI*** | **0.074** | **295** |
| ***WNK4*** | **−0.074** | **296** |
| ***RPL35*** | **−0.074** | **297** |
| ***RSL24D1*** | **−0.074** | **298** |
| ***CYB561*** | **0.074** | **299** |
| ***AK001020*** | **−0.073** | **300** |
| ***LUM*** | **−0.073** | **301** |
| ***ACP5*** | **0.073** | **302** |
| ***HMGA1*** | **0.073** | **303** |
| ***FBP1*** | **−0.073** | **304** |
| ***FTH1*** | **0.073** | **305** |
| ***MELK*** | **0.073** | **306** |
| ***FMOD*** | **−0.072** | **307** |
| ***HBA2*** | **−0.072** | **308** |
| ***GPR172A*** | **0.072** | **309** |
| ***TIGA1*** | **−0.072** | **310** |
| ***GSTM2*** | **0.072** | **311** |
| ***TSPAN9*** | **−0.072** | **312** |
| ***POLB*** | **−0.072** | **313** |
| ***NINJ1*** | **−0.072** | **314** |
| ***RPL5*** | **−0.071** | **315** |
| ***CEBPD*** | **−0.071** | **316** |
| ***ASNS*** | **0.071** | **317** |
| ***RBP1*** | **−0.071** | **318** |
| ***UBA1*** | **0.071** | **319** |
| ***AKT1*** | **0.071** | **320** |
| ***DARC*** | **−0.071** | **321** |
| ***RERG*** | **−0.071** | **322** |
| ***PALLD*** | **−0.071** | **323** |
| ***OMD*** | **−0.071** | **324** |
| ***DCN*** | **−0.071** | **325** |
| ***CCNB2*** | **0.071** | **326** |
| ***COL9A2*** | **−0.071** | **327** |
| ***SRPX*** | **−0.071** | **328** |
| ***CTDSPL*** | **−0.071** | **329** |
| ***ARHGEF2*** | **−0.070** | **330** |
| ***RPS27*** | **−0.070** | **331** |
| ***THBS2*** | **−0.070** | **332** |
| ***HSPD1*** | **0.070** | **333** |
| ***ARHGDIA*** | **0.070** | **334** |
| ***ANKRD30A*** | **−0.070** | **335** |
| ***PTRF*** | **−0.070** | **336** |
| ***FOXO3*** | **−0.070** | **337** |
| ***GIPC1*** | **0.070** | **338** |
| ***CHPT1*** | **−0.070** | **339** |
| ***IL17RB*** | **−0.070** | **340** |
| ***SYBU*** | **−0.070** | **341** |
| ***TUFT1*** | **0.070** | **342** |
| ***CCDC25*** | **−0.070** | **343** |
| ***ARID5B*** | **−0.070** | **344** |
| ***IGFBP4*** | **−0.070** | **345** |
| ***SPDEF*** | **0.070** | **346** |
| ***VDAC2*** | **0.070** | **347** |
| ***DUSP1*** | **−0.070** | **348** |
| ***FOS*** | **−0.069** | **349** |
| ***COPG*** | **0.069** | **350** |
