# Supplementary material for: A key genomic subtype associated with lymphovascular invasion in invasive breast cancer
Source: Br J Cancer. 2019 May 22;120(12):1129–36. doi: 10.1038/s41416-019-0486-6 (PMC6738092; doi:10.1038/s41416-019-0486-6)
Supplement: Supplementary file 2 — List of top 350 genes significantly associated with lymphovascular invasion in the remaining METABRIC cases [file 41416_2019_486_MOESM2_ESM.docx]

**Supplementary Table 2. List of top 350 genes significantly associated with lymphovascular invasion in the remaining METABRIC cases**

| **Genes** | **WAD value** | **WAD ranking** |
| --- | --- | --- |
| ***ERBB2*** | **0.191** | **1** |
| ***SERPINA3*** | **−0.170** | **2** |
| ***S100P*** | **0.145** | **3** |
| ***TFF3*** | **0.141** | **4** |
| ***PIP*** | **−0.123** | **5** |
| ***MGP*** | **−0.120** | **6** |
| ***ACTG2*** | **−0.120** | **7** |
| ***GSTP1*** | **−0.120** | **8** |
| ***HBB*** | **−0.119** | **9** |
| ***UBE2C*** | **0.116** | **10** |
| ***MMP11*** | **0.115** | **11** |
| ***ACTG1*** | **−0.112** | **12** |
| ***FOS*** | **−0.107** | **13** |
| ***LY6E*** | **0.107** | **14** |
| ***CNTNAP2*** | **0.106** | **15** |
| ***HBA2*** | **−0.105** | **16** |
| ***YWHAZ*** | **0.104** | **17** |
| ***FLJ40504*** | **0.104** | **18** |
| ***CLIC6*** | **−0.102** | **19** |
| ***PGAP3*** | **0.101** | **20** |
| ***SFRP1*** | **−0.099** | **21** |
| ***HLA-A*** | **−0.097** | **22** |
| ***SPDEF*** | **0.096** | **23** |
| ***CYP4X1*** | **−0.094** | **24** |
| ***STARD10*** | **0.093** | **25** |
| ***SORD*** | **0.093** | **26** |
| ***LTF*** | **−0.092** | **27** |
| ***STC2*** | **−0.092** | **28** |
| ***C19orf33*** | **0.092** | **29** |
| ***SCD*** | **0.091** | **30** |
| ***ATP5E*** | **0.089** | **31** |
| ***abParts*** | **−0.089** | **32** |
| ***CFD*** | **−0.089** | **33** |
| ***SELM*** | **−0.087** | **34** |
| ***X64709*** | **0.087** | **35** |
| ***TOP2A*** | **0.087** | **36** |
| ***MX1*** | **0.086** | **37** |
| ***FCGBP*** | **−0.086** | **38** |
| ***VTCN1*** | **−0.086** | **39** |
| ***KRT17*** | **−0.086** | **40** |
| ***GSTM2*** | **−0.086** | **41** |
| ***CALML5*** | **0.085** | **42** |
| ***KRT8*** | **0.084** | **43** |
| ***NQO1*** | **0.084** | **44** |
| ***SUSD3*** | **−0.084** | **45** |
| ***TMBIM6*** | **0.083** | **46** |
| ***EEF1G*** | **−0.083** | **47** |
| ***DUSP1*** | **−0.082** | **48** |
| ***CXCL12*** | **−0.082** | **49** |
| ***ANXA1*** | **−0.082** | **50** |
| ***NFIX*** | **−0.081** | **51** |
| ***BOLA2B*** | **0.080** | **52** |
| ***HSPB1*** | **0.080** | **53** |
| ***FOXC1*** | **−0.080** | **54** |
| ***FAM83H*** | **0.079** | **55** |
| ***C10orf116*** | **0.078** | **56** |
| ***STAT1*** | **0.078** | **57** |
| ***NUSAP1*** | **0.078** | **58** |
| ***MYH11*** | **−0.078** | **59** |
| ***S100A16*** | **0.077** | **60** |
| ***PSMB3*** | **0.077** | **61** |
| ***GINS2*** | **0.076** | **62** |
| ***COL4A5*** | **−0.076** | **63** |
| ***APOC1*** | **0.076** | **64** |
| ***CXCL14*** | **−0.076** | **65** |
| ***KIAA0101*** | **0.076** | **66** |
| ***NME1*** | **0.075** | **67** |
| ***GRB7*** | **0.075** | **68** |
| ***ISG15*** | **0.075** | **69** |
| ***AGR2*** | **0.074** | **70** |
| ***HIST1H2AC*** | **−0.073** | **71** |
| ***CFB*** | **−0.073** | **72** |
| ***PTTG1*** | **0.073** | **73** |
| ***FAM129A*** | **−0.073** | **74** |
| ***DCN*** | **−0.072** | **75** |
| ***GPR172A*** | **0.072** | **76** |
| ***ATP9A*** | **0.072** | **77** |
| ***CLEC3A*** | **0.072** | **78** |
| ***KRT18*** | **0.071** | **79** |
| ***GAS1*** | **−0.071** | **80** |
| ***APOE*** | **0.071** | **81** |
| ***TPM1*** | **−0.071** | **82** |
| ***MFAP4*** | **−0.070** | **83** |
| ***UCP2*** | **0.070** | **84** |
| ***SPP1*** | **−0.070** | **85** |
| ***FBLN1*** | **−0.070** | **86** |
| ***CDC20*** | **0.069** | **87** |
| ***C8orf55*** | **0.069** | **88** |
| ***FST*** | **−0.068** | **89** |
| ***DKK3*** | **−0.068** | **90** |
| ***PAM*** | **−0.068** | **91** |
| ***NME4*** | **0.068** | **92** |
| ***ZAK*** | **−0.068** | **93** |
| ***UBE2S*** | **0.067** | **94** |
| ***MFGE8*** | **−0.066** | **95** |
| ***PUF60*** | **0.066** | **96** |
| ***MT1X*** | **−0.066** | **97** |
| ***EGR1*** | **−0.066** | **98** |
| ***TUBA1B*** | **0.065** | **99** |
| ***CDCA5*** | **0.065** | **100** |
| ***NAT1*** | **−0.064** | **101** |
| ***SRP9*** | **−0.064** | **102** |
| ***VIM*** | **−0.064** | **103** |
| ***PDLIM1*** | **−0.064** | **104** |
| ***EEF1A2*** | **0.064** | **105** |
| ***SQLE*** | **0.064** | **106** |
| ***DPYSL2*** | **−0.064** | **107** |
| ***COL16A1*** | **−0.064** | **108** |
| ***SLC9A3R1*** | **0.063** | **109** |
| ***NAPRT1*** | **0.063** | **110** |
| ***RRM1*** | **0.063** | **111** |
| ***HIST1H4C*** | **0.063** | **112** |
| ***SLC40A1*** | **−0.063** | **113** |
| ***PPAP2B*** | **−0.063** | **114** |
| ***EZR*** | **0.062** | **115** |
| ***CYC1*** | **0.062** | **116** |
| ***BST2*** | **0.062** | **117** |
| ***WWP1*** | **0.062** | **118** |
| ***STC1*** | **−0.062** | **119** |
| ***JUN*** | **−0.062** | **120** |
| ***SRPX*** | **−0.062** | **121** |
| ***RPS26*** | **0.062** | **122** |
| ***SERPINE2*** | **−0.061** | **123** |
| ***TMEM97*** | **0.061** | **124** |
| ***PRC1*** | **0.061** | **125** |
| ***TNC*** | **−0.061** | **126** |
| ***CMTM7*** | **−0.061** | **127** |
| ***CITED4*** | **−0.061** | **128** |
| ***SEZ6L2*** | **−0.061** | **129** |
| ***TSC22D1*** | **−0.061** | **130** |
| ***IDH2*** | **0.061** | **131** |
| ***HNRNPA1L2*** | **−0.060** | **132** |
| ***38777*** | **0.060** | **133** |
| ***S100A4*** | **−0.060** | **134** |
| ***VPS28*** | **0.060** | **135** |
| ***ZFP36*** | **−0.060** | **136** |
| ***CCDC130*** | **−0.059** | **137** |
| ***SLC44A1*** | **−0.059** | **138** |
| ***CD24*** | **−0.058** | **139** |
| ***EIF3E*** | **0.058** | **140** |
| ***PRNP*** | **−0.058** | **141** |
| ***PLAT*** | **−0.058** | **142** |
| ***MAL2*** | **0.058** | **143** |
| ***DDIT4*** | **0.058** | **144** |
| ***CGNL1*** | **−0.058** | **145** |
| ***PDGFRL*** | **−0.058** | **146** |
| ***ITM2A*** | **−0.058** | **147** |
| ***ARL6IP1*** | **0.058** | **148** |
| ***KRT19*** | **0.057** | **149** |
| ***CRIP1*** | **0.057** | **150** |
| ***NINJ1*** | **−0.057** | **151** |
| ***TSPAN13*** | **0.057** | **152** |
| ***CPNE3*** | **0.057** | **153** |
| ***ECHDC2*** | **−0.057** | **154** |
| ***CSE1L*** | **0.057** | **155** |
| ***GLA*** | **−0.057** | **156** |
| ***SLC7A2*** | **−0.057** | **157** |
| ***CUEDC1*** | **0.056** | **158** |
| ***EEF1B2*** | **−0.056** | **159** |
| ***PRDX1*** | **0.056** | **160** |
| ***BCAS4*** | **0.056** | **161** |
| ***TUBB2B*** | **−0.056** | **162** |
| ***CSTB*** | **−0.056** | **163** |
| ***ATP5EP2*** | **0.055** | **164** |
| ***PGM1*** | **−0.055** | **165** |
| ***GSDMB*** | **0.055** | **166** |
| ***FSCN1*** | **−0.055** | **167** |
| ***SGCE*** | **−0.055** | **168** |
| ***STK3*** | **0.055** | **169** |
| ***TM7SF2*** | **0.055** | **170** |
| ***EIF2C2*** | **0.055** | **171** |
| ***PABPC1*** | **0.055** | **172** |
| ***FGD3*** | **−0.054** | **173** |
| ***RBBP8*** | **−0.054** | **174** |
| ***NCOA3*** | **0.054** | **175** |
| ***PBX3*** | **−0.054** | **176** |
| ***ORMDL3*** | **0.054** | **177** |
| ***MAOA*** | **−0.054** | **178** |
| ***ANG*** | **−0.053** | **179** |
| ***SERHL2*** | **0.053** | **180** |
| ***FBLN2*** | **−0.053** | **181** |
| ***C9orf46*** | **−0.053** | **182** |
| ***MMP7*** | **−0.053** | **183** |
| ***TMEM106C*** | **0.053** | **184** |
| ***ALCAM*** | **0.053** | **185** |
| ***CCNB2*** | **0.053** | **186** |
| ***KRT7*** | **0.053** | **187** |
| ***SGK223*** | **−0.053** | **188** |
| ***MFSD3*** | **0.053** | **189** |
| ***ALOX5*** | **−0.053** | **190** |
| ***ALOX5AP*** | **−0.053** | **191** |
| ***CA2*** | **−0.053** | **192** |
| ***ATP6V0B*** | **0.052** | **193** |
| ***FGFR3*** | **0.052** | **194** |
| ***APOD*** | **−0.052** | **195** |
| ***TGOLN2*** | **−0.052** | **196** |
| ***ZDHHC8*** | **−0.052** | **197** |
| ***ZFP36L2*** | **−0.052** | **198** |
| ***MYC*** | **−0.052** | **199** |
| ***NCRNA00152*** | **0.052** | **200** |
| ***PCOLCE*** | **−0.052** | **201** |
| ***RPL19*** | **0.052** | **202** |
| ***MCM4*** | **0.052** | **203** |
| ***NTN4*** | **−0.052** | **204** |
| ***FOSB*** | **−0.052** | **205** |
| ***LASS6*** | **0.052** | **206** |
| ***EIF3G*** | **−0.052** | **207** |
| ***CKMT1B*** | **0.052** | **208** |
| ***COL6A1*** | **−0.051** | **209** |
| ***TMEM14C*** | **−0.051** | **210** |
| ***CST3*** | **−0.051** | **211** |
| ***RPL3*** | **−0.051** | **212** |
| ***SLC38A1*** | **0.051** | **213** |
| ***FRMD6*** | **−0.051** | **214** |
| ***SLC5A6*** | **0.051** | **215** |
| ***TNS3*** | **−0.051** | **216** |
| ***C1S*** | **−0.051** | **217** |
| ***PLSCR3*** | **−0.051** | **218** |
| ***CANT1*** | **0.050** | **219** |
| ***PTPN1*** | **0.050** | **220** |
| ***SC5DL*** | **−0.050** | **221** |
| ***ITM2B*** | **−0.050** | **222** |
| ***MYL6*** | **0.050** | **223** |
| ***IL17RB*** | **−0.050** | **224** |
| ***MT1E*** | **−0.050** | **225** |
| ***CSDA*** | **−0.050** | **226** |
| ***DNAJA4*** | **0.050** | **227** |
| ***TNFSF10*** | **−0.050** | **228** |
| ***NDP*** | **−0.049** | **229** |
| ***C12orf44*** | **0.049** | **230** |
| ***SERF2*** | **0.049** | **231** |
| ***CDC42EP4*** | **−0.049** | **232** |
| ***CYP4Z1*** | **−0.049** | **233** |
| ***LOC389493*** | **−0.049** | **234** |
| ***ADM*** | **−0.049** | **235** |
| ***TMEM101*** | **−0.049** | **236** |
| ***HERPUD1*** | **−0.049** | **237** |
| ***DENND1B*** | **0.049** | **238** |
| ***IFI44L*** | **0.049** | **239** |
| ***MRPL27*** | **0.049** | **240** |
| ***ALPL*** | **−0.049** | **241** |
| ***WLS*** | **−0.049** | **242** |
| ***CXCL10*** | **0.049** | **243** |
| ***ARMCX1*** | **−0.049** | **244** |
| ***KRT15*** | **−0.049** | **245** |
| ***LAPTM4B*** | **0.049** | **246** |
| ***CLDN3*** | **0.049** | **247** |
| ***ZBTB20*** | **−0.049** | **248** |
| ***COPS5*** | **0.048** | **249** |
| ***DNAJC12*** | **−0.048** | **250** |
| ***ID3*** | **−0.048** | **251** |
| ***UBE2E3*** | **−0.048** | **252** |
| ***PITX1*** | **0.048** | **253** |
| ***GAPDH*** | **0.048** | **254** |
| ***HLA-B*** | **−0.048** | **255** |
| ***SDCBP*** | **−0.048** | **256** |
| ***LRRC26*** | **0.048** | **257** |
| ***TNFRSF14*** | **−0.048** | **258** |
| ***CRTAP*** | **−0.048** | **259** |
| ***C8orf4*** | **−0.048** | **260** |
| ***NOSTRIN*** | **−0.048** | **261** |
| ***GNAS*** | **0.047** | **262** |
| ***UBD*** | **−0.047** | **263** |
| ***FAM127A*** | **−0.047** | **264** |
| ***CHI3L2*** | **−0.047** | **265** |
| ***GATA3*** | **−0.047** | **266** |
| ***AURKA*** | **0.047** | **267** |
| ***SCPEP1*** | **−0.047** | **268** |
| ***TXNIP*** | **−0.047** | **269** |
| ***ZNF148*** | **0.047** | **270** |
| ***QPCT*** | **−0.047** | **271** |
| ***CD248*** | **−0.047** | **272** |
| ***PRDX2*** | **−0.047** | **273** |
| ***BOLA2*** | **0.047** | **274** |
| ***GRINA*** | **0.047** | **275** |
| ***hNp95*** | **0.047** | **276** |
| ***COX6C*** | **0.047** | **277** |
| ***RPL30*** | **0.047** | **278** |
| ***IGJ*** | **−0.047** | **279** |
| ***TGFBR2*** | **−0.047** | **280** |
| ***STIP1*** | **0.047** | **281** |
| ***TDG*** | **0.047** | **282** |
| ***KRT6B*** | **−0.047** | **283** |
| ***CLN3*** | **0.047** | **284** |
| ***PTGDS*** | **−0.046** | **285** |
| ***NOP56*** | **0.046** | **286** |
| ***ELF3*** | **0.046** | **287** |
| ***ASAP1*** | **0.046** | **288** |
| ***C8orf84*** | **−0.046** | **289** |
| ***SLC1A5*** | **0.046** | **290** |
| ***MLPH*** | **0.046** | **291** |
| ***KIAA0182*** | **0.046** | **292** |
| ***FCER1A*** | **−0.046** | **293** |
| ***BZW2*** | **0.046** | **294** |
| ***MAPT*** | **−0.046** | **295** |
| ***GSN*** | **−0.046** | **296** |
| ***TMED3*** | **0.046** | **297** |
| ***HMGA1*** | **0.046** | **298** |
| ***ATP5H*** | **0.046** | **299** |
| ***CSNK1E*** | **−0.046** | **300** |
| ***CAMK2N1*** | **0.046** | **301** |
| ***ERGIC1*** | **0.046** | **302** |
| ***CR613620*** | **0.045** | **303** |
| ***ENPP5*** | **−0.045** | **304** |
| ***GGCT*** | **0.045** | **305** |
| ***C17orf97*** | **−0.045** | **306** |
| ***CAPS*** | **0.045** | **307** |
| ***KIAA1598*** | **0.045** | **308** |
| ***SERPINA1*** | **−0.045** | **309** |
| ***RPS19*** | **0.045** | **310** |
| ***SLC39A11*** | **0.045** | **311** |
| ***SAT1*** | **−0.045** | **312** |
| ***ACTB*** | **−0.045** | **313** |
| ***NUCB1*** | **−0.045** | **314** |
| ***SEMA6A*** | **−0.045** | **315** |
| ***CRISPLD2*** | **−0.045** | **316** |
| ***TMEM62*** | **0.045** | **317** |
| ***CYBRD1*** | **−0.045** | **318** |
| ***MT1G*** | **−0.045** | **319** |
| ***PTTG3*** | **0.045** | **320** |
| ***GADD45A*** | **−0.045** | **321** |
| ***RNASE1*** | **−0.045** | **322** |
| ***PYCARD*** | **−0.045** | **323** |
| ***LPIN1*** | **−0.045** | **324** |
| ***PPIC*** | **−0.045** | **325** |
| ***DQ893812*** | **−0.045** | **326** |
| ***HMGB3*** | **0.045** | **327** |
| ***ZHX1*** | **0.044** | **328** |
| ***NUDT1*** | **0.044** | **329** |
| ***POLR3GL*** | **−0.044** | **330** |
| ***TP53INP1*** | **0.044** | **331** |
| ***TUFM*** | **0.044** | **332** |
| ***CEBPD*** | **−0.044** | **333** |
| ***IFI27*** | **0.044** | **334** |
| ***SOX18*** | **−0.044** | **335** |
| ***RACGAP1*** | **0.044** | **336** |
| ***ST3GAL1*** | **0.044** | **337** |
| ***H2AFX*** | **0.044** | **338** |
| ***PTK2*** | **0.044** | **339** |
| ***SNX3*** | **−0.044** | **340** |
| ***CCDC92*** | **−0.044** | **341** |
| ***AK001020*** | **−0.044** | **342** |
| ***FAM110A*** | **0.044** | **343** |
| ***SCGB1D2*** | **−0.044** | **344** |
| ***IFIT1*** | **0.044** | **345** |
| ***FKBP9L*** | **−0.044** | **346** |
| ***HLA-DQA1*** | **−0.044** | **347** |
| ***TPM2*** | **−0.043** | **348** |
| ***CDS1*** | **0.043** | **349** |
| ***CLIP3*** | **−0.043** | **350** |
