# Supplementary material for: A key genomic subtype associated with lymphovascular invasion in invasive breast cancer
Source: Br J Cancer. 2019 May 22;120(12):1129–36. doi: 10.1038/s41416-019-0486-6 (PMC6738092; doi:10.1038/s41416-019-0486-6)
Supplement: Supplementary file 3 — Correlation between lymphovascular invasion and clinicopathological characteristics [file 41416_2019_486_MOESM3_ESM.docx]

**Supplementary Table 3. Correlation between lymphovascular invasion and clinicopathological characteristics**

| **METABRIC cohort** | | | | | | **TCGA cohort** | | | | | |
| --- | --- | --- | --- | --- | --- | --- | --- | --- | --- | --- | --- |
| **Factors** | | **LVI status** | | | ***p*-value** | **Factors** | | **LVI status** | | | ***p*-value** |
|  |  | **Positive** | **Negative** | **Total** |  |  |  | **Positive** | **Negative** | **Total** |  |
| **Tumour size** | **> 2cm** | 485 (76.5%) | 582 (63.7%) | 1067 | <0.0001 | **Tumour size** | **T 2-4** | 234 (79.3%) | 381 (68.2%) | 615 | 0.00055 |
|  | **< 2cm** | 149 (23.5%) | 332 (36.3%) | 481 |  |  | **T 1** | 61 (20.7%) | 178 (31.8%) | 239 |  |
| **Nodal status** | **Positive** | 430 (67.8%) | 305 (32.9%) | 735 | <0.0001 | **Nodal status** | **Positive** | 226 (77.1%) | 197 (35.4%) | 423 | <0.0001 |
|  | **Negative** | 204 (32.2%) | 621 (67.1%) | 825 |  |  | **Negative** | 67 (22.9%) | 359 (64.6%) | 426 |  |
| **Histological grade** | **Grade 3** | 374 (60.7%) | 399 (44.6%) | 773 | <0.0001 | **Histological grade** | **Grade 3** | 155 (55.0%) | 197 (36.9%) | 352 | <0.0001 |
|  | **Grade 1, 2** | 242 (39.3%) | 496 (55.4%) | 738 |  |  | **Grade 1, 2** | 127 (45.0%) | 337 (63.1%) | 464 |  |
| **ER** | **Positive** | 473 (74.5%) | 731 (78.6%) | 1204 | 0.058 | **ER** | **Positive** | 219 (76.6%) | 420 (78.1%) | 639 | 0.63 |
|  | **Negative** | 162 (25.5%) | 199 (21.4%) | 361 |  |  | **Negative** | 67(23.4%) | 118 (21.9%) | 185 |  |
| **PR** | **Positive** | 319 (50.2%) | 509 (54.7%) | 828 | 0.080 | **PR** | **Positive** | 194 (68.6%) | 352 (65.8%) | 546 | 0.43 |
|  | **Negative** | 316 (49.8%) | 421 (45.3%) | 737 |  |  | **Negative** | 89 (31.4%) | 183 (34.2%) | 272 |  |
| **HER2** | **Positive** | 105 (16.5%) | 83 (8.9%) | 188 | <0.0001 | **HER2** | **Positive** | 50 (21.2%) | 83 (17.9%) | 133 | 0.29 |
|  | **Negative** | 530 (83.5%) | 847 (91.1%) | 1377 |  |  | **Negative** | 186 (78.8%) | 381 (82.1%) | 567 |  |
| **Molecular subtypes** | **Luminal A** | 225 (35.5%) | 368 (39.7%) | 593 | 0.0021 |  | | | | | |
|  | **Luminal B** | 178 (28.1%) | 215 (23.2%) | 393 |  |  |  |  |  |  |  |
|  | **HER2-enriched** | 87 (13.7%) | 94 (10.1%) | 181 |  |  |  |  |  |  |  |
|  | **Basal-like** | 99 (15.6%) | 147 (15.8%) | 246 |  |  |  |  |  |  |  |
|  | **Normal-like** | 44 (7.0%) | 104 (11.2%) | 148 |  |  |  |  |  |  |  |
| Abbreviations: ER, Oestrogen receptor; PR, Progesterone receptor; LVI, Lymphovascular invasion. | | | | | | | | | | | |
