# Supplementary material for: A key genomic subtype associated with lymphovascular invasion in invasive breast cancer
Source: Br J Cancer. 2019 May 22;120(12):1129–36. doi: 10.1038/s41416-019-0486-6 (PMC6738092; doi:10.1038/s41416-019-0486-6)
Supplement: Supplementary file 4 — Survival analysis based on clinicopathological characteristics including lymphovascular invasion [file 41416_2019_486_MOESM4_ESM.docx]

**Supplementary Table 4. Survival analysis based on clinicopathological characteristics including lymphovascular invasion**

| **METABRIC cohort** | | | | | | | | **TCGA cohort** | | | | | | | |
| --- | --- | --- | --- | --- | --- | --- | --- | --- | --- | --- | --- | --- | --- | --- | --- |
| **Factors** | | **Univariate analysis** | | | **Multivariate analysis** | | | **Factors** | | **Univariate analysis** | | | **Multivariate analysis** | | |
|  |  | **Hazard Ratio** | **95% CI** | ***p*-value** | **Hazard Ratio** | **95% CI** | ***p*-value** |  |  | **Hazard Ratio** | **95% CI** | ***p*-value** | **Hazard Ratio** | **95% CI** | ***p*-value** |
| **LVI** | **Negative** | **Reference** | | | **Reference** | | | **LVI** | **Negative** | **Reference** | | | **Reference** | | |
|  | **Positive** | **1.70** | **1.45-2.01** | **<0.0001** | **1.29** | **1.07-1.56** | **0.0073** |  | **Positive** | **2.22** | **1.46-3.38** | **0.00019** | **2.19** | **1.32-3.62** | **0.0023** |
| **Tumour size** | **< 2cm** | **Reference** | | | **Reference** | | | **Tumour size** | **T1** | **Reference** | | | **Reference** | | |
|  | **> 2cm** | **1.82** | **1.49-2.21** | **<0.0001** | **1.48** | **1.21-1.83** | **0.00018** |  | **T2-4** | **1.81** | **1.08-3.04** | **0.025** | **1.33** | **0.77-2.31** | **0.30** |
| **Nodal status** | **Negative** | **Reference** | | | **Reference** | | | **Nodal status** | **Negative** | **Reference** | | | **Reference** | | |
|  | **Positive** | **2.06** | **1.74-2.44** | **<0.0001** | **1.63** | **1.35-1.97** | **<0.0001** |  | **Positive** | **1.85** | **1.20-2.85** | **0.0056** | **1.13** | **0.67-1.92** | **0.65** |
| **Histological grade** | **Grade 1, 2** | **Reference** | | | **Reference** | | | **Histological grade** | **Grade 1, 2** | **Reference** | | | **Reference** | | |
|  | **Grade 3** | **1.63** | **1.37-1.93** | **<0.0001** | **1.16** | **0.96-1.40** | **0.13** |  | **Grade 3** | **1.46** | **0.94-2.25** | **0.092** | **-** | | |
| **ER** | **Positive** | **Reference** | | | **Reference** | | | **ER** | **Positive** | **Reference** | | | **Reference** | | |
|  | **Negative** | **1.66** | **1.38-1.99** | **<0.0001** | **1.14** | **0.91-1.43** | **0.25** |  | **Negative** | **1.89** | **1.19-2.98** | **0.0065** | **1.70** | **0.82-3.50** | **0.15** |
| **PR** | **Positive** | **Reference** | | | **Reference** | | | **PR** | **Positive** | **Reference** | | | **Reference** | | |
|  | **Negative** | **1.67** | **1.42-1.98** | **<0.0001** | **1.38** | **1.13-1.69** | **0.0020** |  | **Negative** | **1.68** | **1.08-2.61** | **0.020** | **1.21** | **0.60-2.42** | **0.60** |
| **HER2** | **Negative** | **Reference** | | | **Reference** | | | **HER2** | **Negative** | **Reference** | | | **Reference** | | |
|  | **Positive** | **1.92** | **1.54-2.38** | **<0.0001** | **1.45** | **1.15-1.83** | **0.0019** |  | **Positive** | **1.51** | **0.83-2.77** | **0.18** | **-** | | |
| **Abbreviations: ER, Oestrogen receptor; PR, Progesterone receptor; LVI, Lymphovascular invasion.** | | | | | | | | | | | | | | | |
