# Supplementary material for: A key genomic subtype associated with lymphovascular invasion in invasive breast cancer
Source: Br J Cancer. 2019 May 22;120(12):1129–36. doi: 10.1038/s41416-019-0486-6 (PMC6738092; doi:10.1038/s41416-019-0486-6)
Supplement: Supplementary file 5 — Full gene name list of the 99 genes significantly associated with lymphovascular invasion [file 41416_2019_486_MOESM5_ESM.docx]

**Supplementary Table 5. Full gene name list of the 99 genes significantly associated with lymphovascular invasion**

| **Gene symbol** | **Gene name** |
| --- | --- |
| ***ACTG2*** | **actin gamma 2** |
| ***ANG*** | **angiogenin** |
| ***ANXA1*** | **annexin A1** |
| ***APOC1*** | **apolipoprotein C1** |
| ***APOE*** | **apolipoprotein E** |
| ***C1S*** | **complement C1s** |
| ***CALML5*** | **calmodulin-like 5** |
| ***CCNB2*** | **cyclin B2** |
| ***CDC42EP4*** | **CDC42 effector protein 4** |
| ***CDCA5*** | **cell division cycle associated 5** |
| ***CEBPD*** | **CCAAT/enhancer-binding protein delta** |
| ***CFB*** | **complement factor B** |
| ***CFD*** | **complement factor D** |
| ***CLIC6*** | **chloride intracellular channel 6** |
| ***COX6C*** | **cytochrome c oxidase subunit 6C** |
| ***CXCL12*** | **C-X-C motif chemokine ligand 12** |
| ***CXCL14*** | **C-X-C motif chemokine ligand 14** |
| ***CYBRD1*** | **cytochrome b reductase 1** |
| ***CYP4X1*** | **cytochrome P450 family 4 subfamily X member 1** |
| ***DCN*** | **decorin** |
| ***DKK3*** | **dickkopf WNT signaling pathway inhibitor 3** |
| ***DNAJA4*** | **DnaJ heat shock protein family (Hsp40) member A4** |
| ***DPYSL2*** | **dihydropyrimidinase-like 2** |
| ***DUSP1*** | **dual specificity phosphatase 1** |
| ***EEF1A2*** | **eukaryotic translation elongation factor 1 alpha 2** |
| ***EEF1B2*** | **eukaryotic translation elongation factor 1 beta 2** |
| ***ELF3*** | **E74 like ETS transcription factor 3** |
| ***ERBB2*** | **erb-b2 receptor tyrosine kinase 2** |
| ***FBLN1*** | **fibulin-1** |
| ***FCER1A*** | **Fc fragment of IgE receptor Ia** |
| ***FCGBP*** | **Fc fragment of IgG binding protein** |
| ***FGD3*** | **FYVE, RhoGEF and PH domain containing 3** |
| ***FOS*** | **Fos proto-oncogene, AP-1 transcription factor subunit** |
| ***FST*** | **follistatin** |
| ***GAS1*** | **growth arrest specific 1** |
| ***GNAS*** | **GNAS complex locus** |
| ***GSTP1*** | **glutathione S-transferase pi 1** |
| ***HBA2*** | **hemoglobin subunit alpha 2** |
| ***HBB*** | **hemoglobin subunit beta** |
| ***HLA-DQA1*** | **major histocompatibility complex, class II, DQ alpha 1** |
| ***HMGA1*** | **high mobility group AT-hook 1** |
| ***HMGB3*** | **high mobility group box 3** |
| ***HSPB1*** | **heat shock protein family B (small) member 1** |
| ***IDH2*** | **isocitrate dehydrogenase (NADP(+)) 2, mitochondrial** |
| ***IFI27*** | **interferon alpha inducible protein 27** |
| ***IL17RB*** | **interleukin 17 receptor B** |
| ***ISG15*** | **ISG15 ubiquitin-like modifier** |
| ***KRT18*** | **keratin 18** |
| ***KRT18P55*** | **keratin 18 pseudogene 55** |
| ***KRT19*** | **keratin 19** |
| ***KRT7*** | **keratin 7** |
| ***KRT8*** | **keratin 8** |
| ***LAPTM4B*** | **lysosomal protein transmembrane 4 beta** |
| ***LRRC26*** | **leucine rich repeat containing 26** |
| ***LY6E*** | **lymphocyte antigen 6 family member E** |
| ***MAOA*** | **monoamine oxidase A** |
| ***MFAP4*** | **microfibrillar-associated protein 4** |
| ***MGP*** | **matrix Gla protein** |
| ***MMP11*** | **matrix metallopeptidase 11** |
| ***MT1E*** | **metallothionein 1E** |
| ***MX1*** | **MX dynamin like GTPase 1** |
| ***NDP*** | **NDP, norrin cystine knot growth factor** |
| ***NINJ1*** | **ninjurin 1** |
| ***NME1*** | **NME/NM23 nucleoside diphosphate kinase 1** |
| ***NOP56*** | **NOP56 ribonucleoprotein** |
| ***PDGFRL*** | **platelet derived growth factor receptor like** |
| ***PGAP3*** | **post-GPI attachment to proteins 3** |
| ***PITX1*** | **paired like homeodomain 1** |
| ***PLGRKT*** | **plasminogen receptor with a C-terminal lysine** |
| ***PTTG1*** | **pituitary tumor-transforming 1** |
| ***PYCARD*** | **PYD and CARD domain containing** |
| ***RPL3*** | **ribosomal protein L3** |
| ***S100A4*** | **S100 calcium binding protein A4** |
| ***S100P*** | **S100 calcium binding protein P** |
| ***SCD*** | **stearoyl-CoA desaturase** |
| ***SELENOM*** | **selenoprotein M** |
| ***SERPINA3*** | **serpin family A member 3** |
| ***SERPINE2*** | **serpin family E member 2** |
| ***SGCE*** | **sarcoglycan epsilon** |
| ***SLC40A1*** | **solute carrier family 40 member 1** |
| ***SLC44A1*** | **solute carrier family 44 member 1** |
| ***SLC52A2*** | **solute carrier family 52 member 2** |
| ***SLC9A3R1*** | **SLC9A3 regulator 1** |
| ***SPDEF*** | **SAM pointed domain containing ETS transcription factor** |
| ***SRPX*** | **sushi repeat containing protein, X-linked** |
| ***STC2*** | **stanniocalcin 2** |
| ***SUSD3*** | **sushi domain containing 3** |
| ***TM7SF2*** | **transmembrane 7 superfamily member 2** |
| ***TNS3*** | **tensin 3** |
| ***TPM2*** | **tropomyosin 2 (beta)** |
| ***TXNIP*** | **thioredoxin interacting protein** |
| ***UBD*** | **ubiquitin D** |
| ***UBE2C*** | **ubiquitin conjugating enzyme E2 C** |
| ***UBE2S*** | **ubiquitin conjugating enzyme E2 S** |
| ***UCP2*** | **uncoupling protein 2** |
| ***VIM*** | **vimentin** |
| ***VTCN1*** | **V-set domain containing T-cell activation inhibitor 1** |
| ***YWHAZ*** | **tyrosine 3-monooxygenase/tryptophan 5-monooxygenase activation protein zeta** |
| ***ZBTB20*** | **zinc finger and BTB domain containing 20** |
