# Supplementary material for: A key genomic subtype associated with lymphovascular invasion in invasive breast cancer
Source: Br J Cancer. 2019 May 22;120(12):1129–36. doi: 10.1038/s41416-019-0486-6 (PMC6738092; doi:10.1038/s41416-019-0486-6)
Supplement: Supplementary file 6 — Mean value, standard error of the mean (SEM), subtraction and weighted average difference (WAD) ranking in the 99 genes significantly associated with lymphovascular invasion [file 41416_2019_486_MOESM6_ESM.docx]

**Supplementary Table 6. Mean value, standard error of the mean (SEM), subtraction and weighted average difference (WAD) ranking in the 99 genes significantly associated with lymphovascular invasion**

| **Upregulated genes** | | | | | | | | | | | | |
| --- | --- | --- | --- | --- | --- | --- | --- | --- | --- | --- | --- | --- |
|  | **Nottingham cases** | | | | | | **Remaining METABRIC cases** | | | | | |
| **LVI** | **Positive** | | **Negative** | | **Subtraction** | **WAD ranking** | **Positive** | | **Negative** | | **Subtraction** | **WAD ranking** |
| **Genes** | **Mean** | **SEM** | **Mean** | **SEM** |  |  | **Mean** | **SEM** | **Mean** | **SEM** |  |  |
| ***APOC1*** | **10.28** | **0.91** | **10.00** | **0.98** | **0.28** | **25** | **9.98** | **0.99** | **9.84** | **1.04** | **0.14** | **64** |
| ***APOE*** | **11.73** | **0.67** | **11.53** | **0.74** | **0.20** | **31** | **11.64** | **0.82** | **11.54** | **0.82** | **0.10** | **81** |
| ***CALML5*** | **7.56** | **2.05** | **7.04** | **1.88** | **0.52** | **61** | **7.50** | **2.16** | **7.16** | **1.93** | **0.34** | **42** |
| ***CCNB2*** | **8.25** | **0.92** | **8.04** | **0.93** | **0.21** | **326** | **8.07** | **0.90** | **7.90** | **1.01** | **0.16** | **186** |
| ***CDCA5*** | **8.50** | **0.96** | **8.28** | **0.98** | **0.22** | **232** | **8.48** | **0.95** | **8.31** | **1.04** | **0.17** | **100** |
| ***COX6C*** | **12.98** | **0.63** | **12.89** | **0.65** | **0.09** | **263** | **12.89** | **0.68** | **12.83** | **0.67** | **0.05** | **277** |
| ***DNAJA4*** | **9.17** | **0.75** | **8.98** | **0.81** | **0.20** | **205** | **9.20** | **0.82** | **9.09** | **0.81** | **0.11** | **227** |
| ***EEF1A2*** | **9.08** | **2.00** | **8.46** | **1.92** | **0.63** | **3** | **9.05** | **2.09** | **8.90** | **2.04** | **0.15** | **105** |
| ***ELF3*** | **8.69** | **0.73** | **8.49** | **0.86** | **0.20** | **274** | **8.92** | **0.79** | **8.81** | **0.82** | **0.11** | **287** |
| ***ERBB2*** | **10.83** | **1.59** | **10.63** | **1.33** | **0.20** | **65** | **10.92** | **1.46** | **10.62** | **1.22** | **0.30** | **1** |
| ***GNAS*** | **12.75** | **0.45** | **12.62** | **0.41** | **0.13** | **101** | **12.93** | **0.53** | **12.87** | **0.48** | **0.05** | **262** |
| ***HMGA1*** | **8.48** | **0.64** | **8.28** | **0.77** | **0.20** | **303** | **8.50** | **0.75** | **8.38** | **0.77** | **0.12** | **298** |
| ***HMGB3*** | **7.72** | **0.89** | **7.38** | **0.83** | **0.34** | **166** | **7.64** | **0.88** | **7.48** | **0.91** | **0.16** | **327** |
| ***HSPB1*** | **12.26** | **0.73** | **12.07** | **0.74** | **0.19** | **32** | **12.21** | **0.79** | **12.11** | **0.84** | **0.10** | **53** |
| ***IDH2*** | **9.63** | **0.88** | **9.45** | **0.72** | **0.18** | **179** | **9.63** | **0.83** | **9.51** | **0.86** | **0.12** | **131** |
| ***IFI27*** | **11.95** | **1.30** | **11.73** | **1.15** | **0.21** | **24** | **11.63** | **1.40** | **11.57** | **1.35** | **0.06** | **334** |
| ***ISG15*** | **9.74** | **1.32** | **9.54** | **1.35** | **0.20** | **140** | **9.75** | **1.35** | **9.61** | **1.36** | **0.14** | **69** |
| ***KRT18*** | **11.70** | **0.96** | **11.51** | **1.07** | **0.20** | **36** | **11.84** | **1.05** | **11.75** | **1.07** | **0.09** | **79** |
| ***KRT18P55*** | **10.35** | **0.97** | **10.16** | **1.02** | **0.18** | **120** | **10.17** | **1.08** | **9.99** | **1.10** | **0.18** | **18** |
| ***KRT19*** | **12.51** | **1.14** | **12.37** | **1.33** | **0.14** | **88** | **12.58** | **1.22** | **12.51** | **1.26** | **0.07** | **149** |
| ***KRT7*** | **9.45** | **1.44** | **9.27** | **1.35** | **0.18** | **223** | **9.36** | **1.53** | **9.25** | **1.46** | **0.11** | **187** |
| ***KRT8*** | **10.16** | **0.92** | **9.96** | **0.99** | **0.21** | **90** | **10.46** | **0.99** | **10.32** | **1.01** | **0.14** | **43** |
| ***LAPTM4B*** | **10.43** | **1.06** | **10.23** | **0.92** | **0.20** | **85** | **10.18** | **1.21** | **10.09** | **1.13** | **0.09** | **246** |
| ***LRRC26*** | **9.92** | **1.63** | **9.72** | **1.51** | **0.20** | **125** | **9.92** | **1.55** | **9.83** | **1.53** | **0.09** | **257** |
| ***LY6E*** | **10.45** | **1.00** | **10.25** | **0.95** | **0.20** | **72** | **10.62** | **1.03** | **10.45** | **1.00** | **0.17** | **14** |
| ***MMP11*** | **10.53** | **1.39** | **10.38** | **1.50** | **0.15** | **184** | **10.47** | **1.36** | **10.27** | **1.52** | **0.19** | **11** |
| ***MX1*** | **11.07** | **1.30** | **10.75** | **1.30** | **0.32** | **9** | **11.17** | **1.32** | **11.05** | **1.29** | **0.13** | **37** |
| ***NME1*** | **11.55** | **0.73** | **11.43** | **0.71** | **0.12** | **217** | **11.31** | **0.72** | **11.20** | **0.67** | **0.11** | **67** |
| ***NOP56*** | **9.75** | **0.47** | **9.59** | **0.48** | **0.16** | **242** | **9.95** | **0.56** | **9.87** | **0.58** | **0.09** | **286** |
| ***PGAP3*** | **8.91** | **1.39** | **8.69** | **1.15** | **0.21** | **199** | **8.81** | **1.26** | **8.56** | **0.95** | **0.25** | **20** |
| ***PITX1*** | **9.29** | **1.55** | **8.84** | **1.60** | **0.45** | **11** | **9.34** | **1.59** | **9.23** | **1.61** | **0.10** | **253** |
| ***PTTG1*** | **9.29** | **0.87** | **9.12** | **0.94** | **0.17** | **257** | **9.10** | **0.91** | **8.93** | **1.01** | **0.16** | **73** |
| ***S100P*** | **9.70** | **2.32** | **9.26** | **2.31** | **0.45** | **5** | **9.52** | **2.34** | **9.22** | **2.24** | **0.30** | **3** |
| ***SCD*** | **10.88** | **0.97** | **10.75** | **0.92** | **0.14** | **200** | **10.92** | **1.11** | **10.78** | **1.03** | **0.14** | **30** |
| ***SLC52A2*** | **9.18** | **0.61** | **9.02** | **0.63** | **0.16** | **309** | **9.29** | **0.72** | **9.13** | **0.67** | **0.16** | **76** |
| ***SLC9A3R1*** | **10.77** | **1.04** | **10.59** | **1.01** | **0.18** | **108** | **10.97** | **0.98** | **10.87** | **1.02** | **0.10** | **109** |
| ***SPDEF*** | **9.48** | **1.39** | **9.34** | **1.46** | **0.14** | **346** | **9.74** | **1.34** | **9.55** | **1.45** | **0.19** | **23** |
| ***TM7SF2*** | **8.70** | **0.93** | **8.44** | **0.88** | **0.26** | **132** | **8.70** | **0.95** | **8.56** | **0.91** | **0.14** | **170** |
| ***UBE2C*** | **9.27** | **1.09** | **9.03** | **1.16** | **0.24** | **106** | **9.25** | **1.17** | **8.99** | **1.31** | **0.25** | **10** |
| ***UBE2S*** | **9.29** | **0.71** | **9.02** | **0.73** | **0.27** | **70** | **9.35** | **0.84** | **9.21** | **0.85** | **0.14** | **94** |
| ***UCP2*** | **8.93** | **0.91** | **8.71** | **0.90** | **0.22** | **189** | **9.10** | **0.94** | **8.94** | **0.92** | **0.16** | **84** |
| ***YWHAZ*** | **12.00** | **0.59** | **11.84** | **0.58** | **0.15** | **79** | **12.08** | **0.63** | **11.95** | **0.62** | **0.13** | **17** |
| **Downregulated genes** | | | | | | | | | | | | |
|  | **Nottingham cases** | | | | | | **Remaining METABRIC cases** | | | | | |
| **LVI** | **Positive** | | **Negative** | | **Subtraction** | **WAD ranking** | **Positive** | | **Negative** | | **Subtraction** | **WAD ranking** |
| **Genes** | **Mean** | **SEM** | **Mean** | **SEM** |  |  | **Mean** | **SEM** | **Mean** | **SEM** |  |  |
| ***ACTG2*** | **8.75** | **2.50** | **9.01** | **1.81** | **-0.26** | **100** | **8.48** | **1.62** | **8.78** | **1.63** | **-0.30** | **7** |
| ***ANG*** | **8.19** | **0.94** | **8.44** | **1.01** | **-0.25** | **186** | **8.14** | **1.07** | **8.29** | **1.14** | **-0.15** | **179** |
| ***ANXA1*** | **10.91** | **0.68** | **11.08** | **0.71** | **-0.17** | **95** | **10.45** | **0.91** | **10.58** | **0.99** | **-0.13** | **50** |
| ***C1S*** | **10.11** | **0.93** | **10.34** | **0.88** | **-0.24** | **53** | **9.67** | **1.02** | **9.77** | **1.10** | **-0.10** | **217** |
| ***CDC42EP4*** | **10.22** | **0.38** | **10.37** | **0.62** | **-0.15** | **197** | **10.40** | **0.66** | **10.48** | **0.65** | **-0.08** | **232** |
| ***CEBPD*** | **10.09** | **0.53** | **10.21** | **0.70** | **-0.13** | **316** | **10.12** | **0.84** | **10.20** | **0.81** | **-0.08** | **333** |
| ***CFB*** | **10.10** | **2.45** | **10.51** | **1.48** | **-0.41** | **4** | **10.42** | **1.70** | **10.54** | **1.64** | **-0.12** | **72** |
| ***CFD*** | **9.48** | **1.39** | **9.87** | **1.38** | **-0.40** | **10** | **9.24** | **1.29** | **9.42** | **1.36** | **-0.19** | **33** |
| ***CLIC6*** | **8.17** | **4.43** | **8.54** | **2.17** | **-0.37** | **50** | **8.15** | **2.21** | **8.43** | **2.24** | **-0.28** | **19** |
| ***CXCL12*** | **9.44** | **1.16** | **9.72** | **1.00** | **-0.28** | **45** | **9.05** | **1.10** | **9.23** | **1.20** | **-0.18** | **49** |
| ***CXCL14*** | **8.31** | **2.30** | **8.67** | **1.52** | **-0.36** | **49** | **8.18** | **1.57** | **8.39** | **1.61** | **-0.21** | **65** |
| ***CYBRD1*** | **9.73** | **1.03** | **9.91** | **1.03** | **-0.18** | **162** | **9.68** | **1.15** | **9.76** | **1.19** | **-0.09** | **318** |
| ***CYP4X1*** | **8.44** | **3.82** | **8.77** | **1.89** | **-0.32** | **64** | **8.65** | **1.90** | **8.88** | **1.94** | **-0.23** | **24** |
| ***DCN*** | **9.07** | **1.34** | **9.23** | **1.24** | **-0.16** | **325** | **8.46** | **1.33** | **8.64** | **1.43** | **-0.19** | **75** |
| ***DKK3*** | **9.45** | **0.94** | **9.72** | **0.88** | **-0.27** | **54** | **9.07** | **0.91** | **9.22** | **0.93** | **-0.15** | **90** |
| ***DPYSL2*** | **9.82** | **0.43** | **9.98** | **0.60** | **-0.16** | **214** | **9.73** | **0.68** | **9.85** | **0.76** | **-0.12** | **107** |
| ***DUSP1*** | **10.32** | **0.90** | **10.44** | **0.96** | **-0.12** | **348** | **9.89** | **1.40** | **10.04** | **1.45** | **-0.15** | **48** |
| ***EEF1B2*** | **11.20** | **0.34** | **11.33** | **0.52** | **-0.12** | **219** | **10.93** | **0.78** | **11.01** | **0.80** | **-0.08** | **159** |
| ***FBLN1*** | **10.59** | **1.04** | **10.86** | **0.93** | **-0.27** | **17** | **10.51** | **1.04** | **10.63** | **1.12** | **-0.11** | **86** |
| ***FCER1A*** | **7.41** | **1.20** | **7.76** | **1.27** | **-0.36** | **144** | **6.95** | **1.07** | **7.15** | **1.22** | **-0.21** | **293** |
| ***FCGBP*** | **8.72** | **2.50** | **9.11** | **1.64** | **-0.39** | **19** | **8.76** | **1.61** | **8.96** | **1.61** | **-0.20** | **38** |
| ***FGD3*** | **8.81** | **1.30** | **9.19** | **1.11** | **-0.38** | **21** | **9.19** | **1.20** | **9.30** | **1.20** | **-0.12** | **173** |
| ***FOS*** | **10.12** | **1.85** | **10.24** | **1.37** | **-0.12** | **349** | **9.53** | **1.66** | **9.74** | **1.69** | **-0.21** | **13** |
| ***FST*** | **8.22** | **1.20** | **8.60** | **1.10** | **-0.38** | **41** | **8.04** | **1.02** | **8.24** | **1.03** | **-0.20** | **89** |
| ***GAS1*** | **8.92** | **1.05** | **9.11** | **0.92** | **-0.19** | **227** | **8.45** | **1.06** | **8.63** | **1.11** | **-0.18** | **80** |
| ***GSTP1*** | **10.76** | **1.21** | **10.99** | **0.93** | **-0.23** | **33** | **10.61** | **1.20** | **10.80** | **1.10** | **-0.19** | **8** |
| ***HBA2*** | **9.40** | **2.26** | **9.55** | **1.48** | **-0.15** | **308** | **9.03** | **1.52** | **9.26** | **1.57** | **-0.23** | **16** |
| ***HBB*** | **9.34** | **2.23** | **9.59** | **1.47** | **-0.24** | **73** | **8.62** | **1.62** | **8.91** | **1.69** | **-0.29** | **9** |
| ***HLA-DQA1*** | **10.37** | **1.03** | **10.52** | **0.99** | **-0.15** | **188** | **10.04** | **1.30** | **10.12** | **1.31** | **-0.08** | **347** |
| ***IL17RB*** | **7.50** | **1.18** | **7.75** | **1.17** | **-0.25** | **340** | **7.56** | **1.06** | **7.73** | **1.04** | **-0.17** | **224** |
| ***MAOA*** | **7.42** | **1.62** | **7.83** | **1.25** | **-0.41** | **84** | **7.48** | **1.33** | **7.67** | **1.37** | **-0.19** | **178** |
| ***MFAP4*** | **8.48** | **1.52** | **8.73** | **1.26** | **-0.25** | **141** | **8.31** | **1.17** | **8.50** | **1.31** | **-0.19** | **83** |
| ***MGP*** | **12.95** | **1.44** | **13.28** | **1.10** | **-0.33** | **2** | **12.73** | **1.34** | **12.87** | **1.43** | **-0.14** | **6** |
| ***MT1E*** | **9.78** | **1.26** | **10.10** | **1.10** | **-0.32** | **15** | **9.75** | **1.23** | **9.84** | **1.19** | **-0.09** | **225** |
| ***NDP*** | **6.81** | **2.19** | **7.26** | **1.53** | **-0.45** | **152** | **6.91** | **1.65** | **7.14** | **1.68** | **-0.23** | **229** |
| ***NINJ1*** | **10.21** | **0.29** | **10.33** | **0.53** | **-0.12** | **314** | **10.40** | **0.55** | **10.49** | **0.53** | **-0.09** | **151** |
| ***PDGFRL*** | **8.95** | **1.04** | **9.23** | **0.95** | **-0.28** | **67** | **8.51** | **1.01** | **8.65** | **1.09** | **-0.15** | **146** |
| ***PLGRKT*** | **9.88** | **0.38** | **10.05** | **0.63** | **-0.18** | **160** | **9.62** | **0.78** | **9.72** | **0.78** | **-0.10** | **182** |
| ***PYCARD*** | **9.90** | **0.83** | **10.10** | **0.88** | **-0.19** | **122** | **10.05** | **0.94** | **10.13** | **0.94** | **-0.08** | **323** |
| ***RPL3*** | **12.76** | **0.29** | **12.89** | **0.46** | **-0.13** | **112** | **12.70** | **0.53** | **12.76** | **0.53** | **-0.06** | **212** |
| ***S100A4*** | **10.87** | **0.71** | **11.00** | **0.76** | **-0.13** | **209** | **10.46** | **0.86** | **10.55** | **0.90** | **-0.10** | **134** |
| ***SELENOM*** | **10.09** | **0.49** | **10.33** | **0.69** | **-0.23** | **55** | **10.20** | **0.70** | **10.35** | **0.66** | **-0.15** | **34** |
| ***SERPINA3*** | **12.09** | **3.18** | **12.25** | **1.79** | **-0.16** | **63** | **12.05** | **1.79** | **12.27** | **1.68** | **-0.21** | **2** |
| ***SERPINE2*** | **9.97** | **0.89** | **10.28** | **0.96** | **-0.31** | **16** | **9.82** | **1.03** | **9.93** | **1.08** | **-0.11** | **123** |
| ***SGCE*** | **8.87** | **0.97** | **9.09** | **0.89** | **-0.21** | **176** | **8.49** | **1.10** | **8.63** | **1.14** | **-0.14** | **168** |
| ***SLC40A1*** | **9.83** | **1.18** | **10.07** | **1.29** | **-0.24** | **59** | **9.71** | **1.32** | **9.83** | **1.40** | **-0.12** | **113** |
| ***SLC44A1*** | **11.03** | **0.25** | **11.24** | **0.50** | **-0.21** | **46** | **10.93** | **0.57** | **11.01** | **0.55** | **-0.09** | **138** |
| ***SRPX*** | **8.24** | **1.02** | **8.43** | **0.93** | **-0.20** | **328** | **7.84** | **1.00** | **8.04** | **1.14** | **-0.19** | **121** |
| ***STC2*** | **9.26** | **3.41** | **9.70** | **1.94** | **-0.44** | **6** | **9.73** | **1.96** | **9.90** | **1.93** | **-0.17** | **28** |
| ***SUSD3*** | **8.46** | **2.30** | **8.99** | **1.55** | **-0.53** | **7** | **8.67** | **1.57** | **8.87** | **1.57** | **-0.20** | **45** |
| ***TNS3*** | **9.84** | **0.32** | **10.04** | **0.50** | **-0.19** | **129** | **9.98** | **0.62** | **10.07** | **0.58** | **-0.09** | **216** |
| ***TPM2*** | **10.48** | **0.74** | **10.61** | **0.75** | **-0.13** | **275** | **10.32** | **0.78** | **10.40** | **0.82** | **-0.07** | **348** |
| ***TXNIP*** | **10.16** | **0.35** | **10.29** | **0.63** | **-0.14** | **259** | **9.92** | **0.72** | **10.00** | **0.76** | **-0.09** | **269** |
| ***UBD*** | **8.16** | **2.50** | **8.55** | **1.66** | **-0.39** | **40** | **7.98** | **1.56** | **8.12** | **1.61** | **-0.14** | **263** |
| ***VIM*** | **12.25** | **0.39** | **12.41** | **0.58** | **-0.16** | **57** | **12.05** | **0.77** | **12.13** | **0.83** | **-0.08** | **103** |
| ***VTCN1*** | **9.12** | **3.46** | **9.34** | **2.00** | **-0.22** | **134** | **9.08** | **2.01** | **9.26** | **1.96** | **-0.19** | **39** |
| ***ZBTB20*** | **8.79** | **0.51** | **8.96** | **0.63** | **-0.17** | **300** | **8.92** | **0.68** | **9.02** | **0.72** | **-0.10** | **342** |
