# Supplementary material for: A key genomic subtype associated with lymphovascular invasion in invasive breast cancer
Source: Br J Cancer. 2019 May 22;120(12):1129–36. doi: 10.1038/s41416-019-0486-6 (PMC6738092; doi:10.1038/s41416-019-0486-6)
Supplement: Supplementary file 7 — Survival analysis based on clinicopathological characteristics including LVI-related genomic subtype [file 41416_2019_486_MOESM7_ESM.docx]

**Supplementary Table 7. Survival analysis based on clinicopathological characteristics including LVI-related genomic subtype**

| **METABRIC cohort** | | | | | **TCGA cohort** | | | | |
| --- | --- | --- | --- | --- | --- | --- | --- | --- | --- |
| **Factors** | | **Multivariate analysis** | | | **Factors** | | **Multivariate analysis** | | |
|  |  | **Hazard Ratio** | **95% CI** | ***p*-value** |  |  | **Hazard Ratio** | **95% CI** | ***p*-value** |
| **LVI related genomic subtype** | **Subtype 1** | **Reference** | | | **LVI related genomic subtype** | **Subtype 1** | **Reference** | | |
|  | **Subtype 2** | **1.32** | **1.07-1.63** | **0.0098** |  | **Subtype 2** | **2.76** | **1.19-6.38** | **0.018** |
| **LVI** | **Negative** | **Reference** | | | **LVI** | **Negative** | **Reference** | | |
|  | **Positive** | **1.29** | **1.07-1.55** | **0.0075** |  | **Positive** | **1.42** | **0.76-2.65** | **0.28** |
| **Tumour size** | **< 2cm** | **Reference** | | | **Tumour size** | **T1** | **Reference** | | |
|  | **> 2cm** | **1.44** | **1.17-1.78** | **0.00055** |  | **T2-4** | **1.27** | **0.67-2.43** | **0.47** |
| **Nodal status** | **Negative** | **Reference** | | | **Nodal status** | **Negative** | **Reference** | | |
|  | **Positive** | **1.64** | **1.36-1.98** | **<0.0001** |  | **Positive** | **1.38** | **0.72-2.63** | **0.33** |
| **Histological grade** | **Grade 1, 2** | **Reference** | | | **Histological grade** | **Grade 1, 2** | **Reference** | | |
|  | **Grade 3** | **1.07** | **0.88-1.31** | **0.49** |  | **Grade 3** | **0.74** | **0.40-1.39** | **0.35** |
| **ER** | **Positive** | **Reference** | | | **ER** | **Positive** | **Reference** | | |
|  | **Negative** | **1.08** | **0.86-1.36** | **0.51** |  | **Negative** | **1.40** | **0.60-3.30** | **0.44** |
| **PR** | **Positive** | **Reference** | | | **PR** | **Positive** | **Reference** | | |
|  | **Negative** | **1.32** | **1.07-1.62** | **0.0095** |  | **Negative** | **0.92** | **0.41-2.08** | **0.84** |
| **HER2** | **Negative** | **Reference** | | | **HER2** | **Negative** | **Reference** | | |
|  | **Positive** | **1.38** | **1.09-1.74** | **0.0074** |  | **Positive** | **1.20** | **0.63-2.27** | **0.58** |
| **Abbreviations: ER, Oestrogen receptor; PR, Progesterone receptor; LVI, Lymphovascular invasion.** | | | | | | | | | |
