# Supplementary figures and images for: A key genomic subtype associated with lymphovascular invasion in invasive breast cancer
Source: Br J Cancer. 2019 May 22;120(12):1129–36. doi: 10.1038/s41416-019-0486-6 (PMC6738092; doi:10.1038/s41416-019-0486-6)

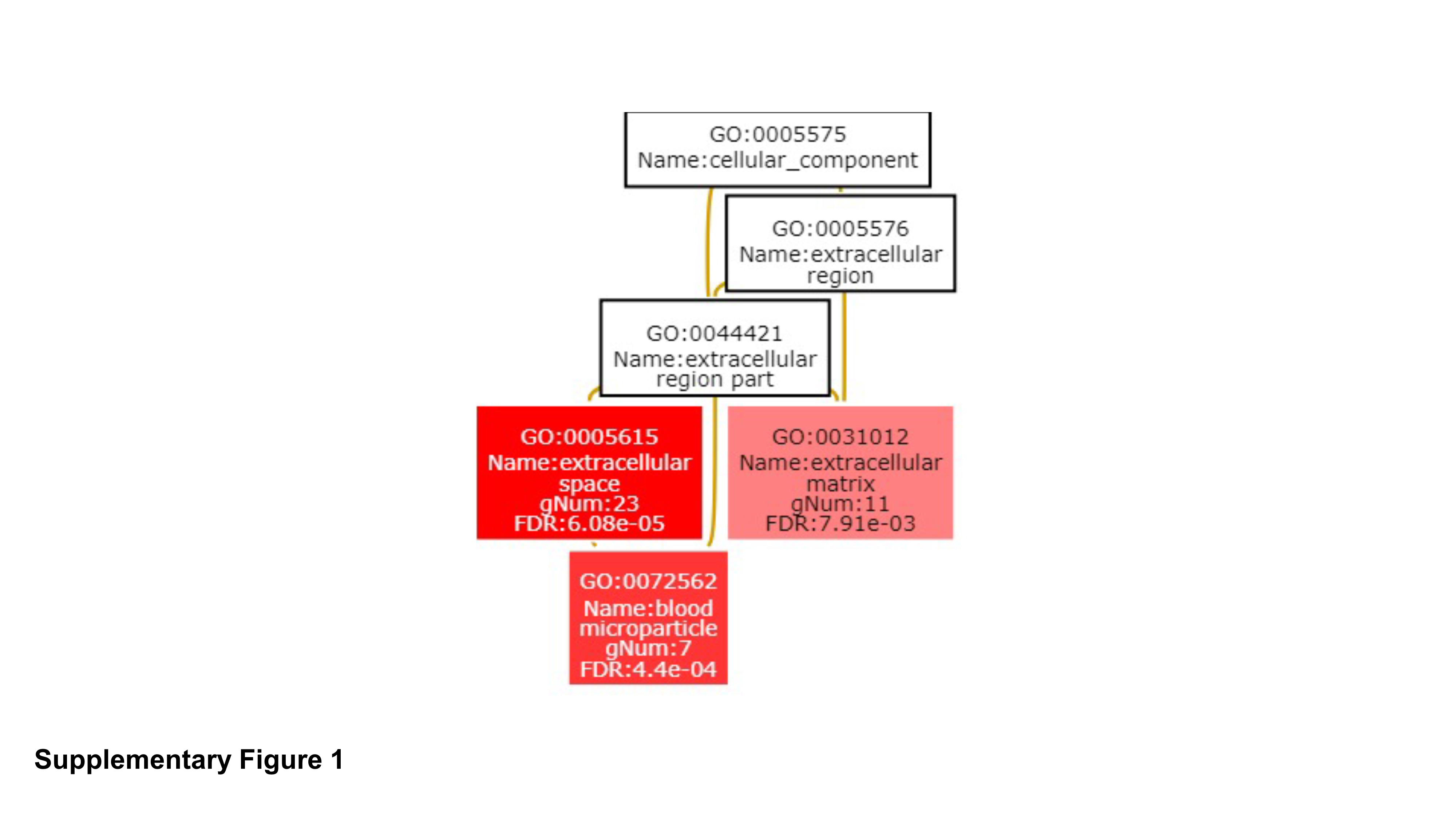

Supplement: Supplementary file 8 — Significant pathways associated with LVI-related gene set [file 41416_2019_486_MOESM8_ESM.tif]
